# Identity negotiation on the LIHKG platform: a grounded theory study of Mainland Chinese immigrants’ adaptation to Hong Kong society

**DOI:** 10.3389/fpsyg.2025.1643942

**Published:** 2025-08-12

**Authors:** Lingxiao Zhang, Tao Shen

**Affiliations:** ^1^City University of Hong Kong School of Creative Media, Hong Kong, Hong Kong SAR, China; ^2^Tongji University College of Design and Innovation, Shanghai, China

**Keywords:** identity negotiation, digital migration, LIHKG platform, Mainland Chinese immigrants, acculturation strategies

## Abstract

In digital societies, social media has emerged as a critical arena for immigrant communities to engage in identity construction, yet there remains limited research on identity negotiation within specific digital platforms in the Chinese context. This study examines how Mainland Chinese immigrants negotiate identity, express emotions, and engage in social interactions on Hong Kong’s LIHKG platform (a locally dominant online forum established in 2016 that serves as Hong Kong’s primary community discussion platform) to adapt to the local socio-cultural environment. The research conceptualizes place as both physical location (Hong Kong as destination) and digital space (LIHKG as virtual locale), exploring how these intersecting spatial dimensions shape identity construction processes. Using grounded theory methodology, we analyzed 800 platform posts and conducted in-depth interviews with 20 Mainland Chinese immigrants. Results reveal a dynamic identity negotiation process characterized by four patterns (integrative, confrontational, collaborative, and avoidance) that immigrants strategically employ across different contexts. Place emerges as a fundamental organizing principle, with immigrants navigating between physical Hong Kong, digital platform spaces, and imagined cultural territories in their identity work. Emotions emerged as critical resources in identity construction, with specific regulation strategies developed to navigate exclusionary experiences. Interactions between immigrants and locals demonstrated significant topic differentiation, with political discussions exhibiting heightened boundaries while professional and everyday topics facilitated collaborative engagement. LIHKG’s platform features—including anonymity mechanisms and voting systems—fundamentally shape these identity expressions and group dynamics. This research contributes to migration studies by incorporating both digital and physical place dimensions into traditional frameworks, integrating emotional sociology, and developing localized theoretical models specific to Hong Kong-Mainland relations. The findings offer implications for digital inclusion policies, platform governance, immigrant support services, and construction of inclusive public discourse across multiple place-based contexts.

## 1 Introduction

### 1.1 Research background

Place, in the context of contemporary migration studies, extends beyond simple geographical location to encompass multiple interconnected spatial dimensions that shape identity formation processes. For this research, we conceptualize place as operating on three analytical levels: first, physical place—Hong Kong as a distinctive geographical and political destination characterized by the “One Country, Two Systems” framework; second, digital place—the LIHKG platform as a virtual locale with its own cultural norms, spatial organization, and community dynamics; and third, cultural place—the imagined territories of belonging that immigrants navigate between Mainland Chinese origins and Hong Kong locality. These place dimensions intersect and mutually constitute each other, creating complex spatial contexts within which identity negotiation occurs.

The significance of place in identity construction becomes particularly pronounced in the Hong Kong-Mainland China context, where physical proximity coexists with significant political, cultural, and social differentiation. Hong Kong’s unique position as a Special Administrative Region creates what we term “proximate otherness”—a spatial condition where geographical closeness paradoxically heightens rather than diminishes cultural boundaries. This spatial complexity is further amplified in digital environments, where virtual place-making practices on platforms like LIHKG both reflect and reshape offline place-based identities.

As a global metropolis and a Special Administrative Region of China (the international city implementing “One Country, Two Systems”), Hong Kong has experienced ongoing structural transformations in its immigrant population. According to data from the Hong Kong Census and Statistics Department, the net inflow of immigrants holding One-way Permits increased from 17,900 in 2021 to 40,800 in 2023, reaching 44,000 in 2024, reflecting a growth rate of 107.5% compared to that in 2022 (Hong Kong Census and Statistics Department, 2021-2024). This population mobility phenomenon not only pertains to urban resource allocation but also profoundly impacts the socio-cultural ecosystem of Hong Kong’s role as a “super-connector.” The physical movement of people across the Hong Kong-Mainland border creates new place-based identities that must be negotiated both in offline spaces and digital environments. Simultaneously, at the individual level, immigrants face multifaceted challenges, including identity formation, emotional expression, and social interactions across multiple place contexts (Wang, 2012; Yu et al., 2013).

In the digital society, social media has emerged as a critical arena for immigrant communities to engage in social interactions and construction of identities within and across place boundaries (Yau et al., 2019). Established in 2016, LIHKG primarily serves Hong Kong’s local residents. Following the 2014 Umbrella Movement, users gradually shifted from HKGolden Forum (due to its restrictive user policies) to LIHKG (Matthew, 2021). Renowned for its anonymity, LIHKG became particularly prominent during the 2019 Anti-Extradition Law Movement, earning a reputation as a “safe haven” for “wild political” expressions, enabling authentic emotional articulation among users within a distinctively Hong Kong digital place (Erni and Zhang, 2022). As a locally dominant online forum in Hong Kong, LIHKG offers a unique lens through which to observe the identity negotiation and acculturation of Mainland Chinese immigrants as they navigate between their places of origin and destination through digital mediation. This platform serves not only as a channel for accessing local information and expressing personal aspirations but also as a native “meme arena” for interacting with local residents and navigating cultural differences across place-based boundaries. A detailed examination of immigrants’ identity practices on LIHKG holds significant implications for understanding the complexities of contemporary immigrant integration within multi-layered place contexts.

Existing research suggests that interactions in virtual spaces often shape immigrants’ cultural adaptation trajectories (Berry, 2005). However, these studies primarily analyze offline social networks of North American immigrant communities, leaving a gap in empirical research on identity construction within specific digital platforms in the Chinese context, particularly in Hong Kong’s high-context society where place-based cultural distinctions remain highly salient. New immigrants from Mainland China to Hong Kong face identity negotiation-centered conflicts with local residents rooted in contested understandings of place-based belonging (Ma and Holford, 2023). Identity negotiation is a multidimensional and complex concept, encompassing cultural adaptation, political factors, historical contexts, and economic status as these intersect with place-based experiences (Bucholtz and Hall, 2005; Hermans and Dimaggio, 2007). This study employs a three-stage coding analysis of 800 LIHKG posts, based on dozens of keywords such as “new Hong Kongers,” “Mainland new immigrants,” and “Mainland drifters.” Observations indicate that LIHKG, as Hong Kong’s most localized social media platform, reflects varying degrees of negative sentiments and non-acceptance among local users toward Mainland new immigrants that are fundamentally organized around place-based categories of belonging. These sentiments stem from factors such as linguistic differences, competition for subsidized housing, medical resources, educational resources, and stereotypical perceptions all of which are tied to contested claims over Hong Kong as place. Additionally, the study includes interviews with 20 Mainland Chinese immigrants who use LIHKG, with the interview data utilized for grounded theory analysis.

### 1.2 Research question

Based on the aforementioned research background, this study proposes the following central question: How do Mainland Chinese immigrants construct identity, express emotional needs, and engage in social interactions through the LIHKG platform to adapt to Hong Kong’s socio-cultural environment across multiple place dimensions? This overarching question is further refined into three sub-questions:

(1) Identity Negotiation: How do Mainland Chinese immigrants adjust their identity in the process of navigating cultural conflicts and adaptation between place-based belonging categories?
(2) Emotional Expression: How do Mainland Chinese immigrants express identity-related emotions on the LIHKG platform as these relate to place-based experiences of inclusion and exclusion?
(3) Group Interaction: How do Mainland Chinese immigrants interact with Hong Kong local residents and other social groups through the LIHKG platform in ways that reflect and reshape place-based boundaries?

These questions collectively form a comprehensive analytical framework for understanding immigrants’ online lives, including multiple dimensions such as identity, emotion, and behavior, and reflecting the dynamic process of immigrant cultural adaptation across intersecting place contexts.

### 1.3 Research objectives, contents and significance

This study takes the LIHKG platform as its research field and comprehensively integrates qualitative research methods such as grounded theory and online ethnography for the purpose of achieving following objectives:

First objective is uncover the processes and mechanisms of identity negotiation among immigrant groups within digital platforms with particular attention to how place-based categories organize these processes; second is to understand the impact of social media interactions on immigrants’ emotional expression and social participation across virtual and physical place contexts; and third is to develop a theory of immigrant acculturation tailored to the Hong Kong context, providing empirical support for promoting social integration in Hong Kong that accounts for the complex place dynamics characterizing this unique spatial context.

To achieve these objectives, the study will proceed as follows: First, it will systematically review literature in the fields of immigrant identity and digital media to clarify the research background and theoretical context. Existing studies indicate a lack of systematic analytical frameworks, highlighting the need to further explore how social media influences immigrants’ identity negotiation processes particularly as these unfold across multiple place dimensions. They also suggest that future research should develop theoretical models to deepen the understanding of dynamic identity negotiation, particularly in the complexity of digital environments where virtual and physical places intersect (Lecheler et al., 2019). Urban influencers, through social media content, construct a “super-diverse identity,” and propose “mediated urban belonging,” which strengthens identity negotiation and group interaction experiences in offline spaces through online content (Van Eldik et al., 2019). Another study, grounded in the “uses and gratifications” theory, identifies three primary motives for user-generated content (UGC), including social group interaction and self-expression, and recommends the enhancement of new immigrants’ emotional expression capabilities through digital platform usage skills (Shao, 2009). Second, the study will deeply analyze the identity discourses of Mainland Chinese immigrants on the LIHKG platform, employing grounded theory coding procedures to distill core categories of identity negotiation. Third, it will examine immigrants’ online interactions within the LIHKG community, and analyze their emotional expression, intergroup communication strategies, and outcomes. Finally, based on the research findings, the study will reflect on the potential for extending traditional immigration theories in digital contexts and provide policy implications for immigrant integration practices in Hong Kong.

## 2 Literature review

Immigrant identity is one of the core issues in cross-cultural research. As globalization accelerates, an increasing number of immigrants cross national borders and reshape their self-identity in new environments. Scholars have engaged in extensive and in-depth discussions on immigrant identity, giving rise to diverse theoretical perspectives. This chapter aims to review the existing research trajectory, with a particular focus on exploring new trends and challenges in immigrant identity construction in the digital era. By integrating the Hong Kong context, it seeks to refine the theoretical questions of this study.

### 2.1 Traditional immigration identity theory

There has been a long history for the study of immigrant identity. Early research primarily approached the topic from the perspective of acculturation, focusing on how immigrants adjust their cultural identification during the migration process. Berry (2005) proposed the widely cited two-dimensional acculturation model and categorized immigrant adaptation strategies into four types: assimilation, integration, separation, and marginalization. This model elucidates the diverse pathways of identity negotiation immigrants undertake when addressing cultural differences and laid a theoretical foundation for understanding cross-cultural adaptation. On the bases of Berry’s model, Phinney (2003) introduced a three-stage theory of ethnic identity development, suggesting that immigrants form mature and positive ethnic identities through exploration and commitment. Wang (2012), focusing on Chinese diaspora, identified four prominent features of transnational identity: intercultural competence, regional reconstruction, diasporic consciousness, and hybrid belonging. Additionally, Yang (2016) examined the cultural identity and sense of belonging among Chinese immigrants in multicultural societies, emphasizing the complexity and fluidity of identity. Zhu (2015) explored identity construction and citizenship learning among Chinese immigrants in Canadian settlement organizations, and revealed the intricate relationship between immigrant identity and civic identity. These studies all provide multifaceted insights into immigrant identity, and highlight its dynamic and diverse nature.

With ongoing further research, scholars have increasingly focused on the complexity and dynamism of immigrant identity. Bhatia and Ram (2009) argue that traditional acculturation theories often assume a holistic, linear developmental model, while overlook the tensions and struggles inherent in the identity construction process. They advocate for a critical perspective to examine immigrants’ cross-cultural experiences, and explore the dynamic processes of continuous negotiation, hybridization, and reconfiguration of cultural identities across different contexts. This perspective provides a new analytical framework for immigrant identity research. By employing social representation theory, Ellis and Bhatia (2018) further contend that immigrant identity emerges from ongoing negotiations within individual-social interactions. While integrating into mainstream society, immigrants simultaneously reshape their cultural representations, resulting in hybrid and boundary-crossing identity formations.

Overall, traditional immigrant identity theories have primarily focused on offline cross-cultural interactions, nevertheless lack systematic examination of identity construction in the digital era. With the advancement of information technology, immigrants increasingly rely on online platforms for information acquisition, social interaction, and emotional expression. The identity negotiation practices emerging from these digital contexts warrant further exploration.

### 2.2 The construction of immigrant identity in digital platform

The rise of digital media has provided new socio-technical conditions for immigrants’ transnational mobility. An increasing number of studies have begun to explore how immigrants leverage digital platforms for cultural adaptation and identity negotiation. McGregor and Siegel (2013) were among the first to outline three key functions of social media as transnational social spaces for immigrants: information dissemination, emotional support, and social connectivity. These three key functions create opportunities for immigrants to meet socio-psychological needs across geographical boundaries. Leurs and Prabhakar (2018) further argue that digital diasporic networks on online platforms help alleviate immigrants’ sense of isolation in foreign lands, fostering a sense of belonging. This aligns with Diminescu’s (2008) assertion that digital media transcends traditional geographical boundaries, and enable immigrants to maintain ties with their home countries through social media and transnational information networks while dynamically construct identities in new social environments. In the Chinese context, Chinese scholars have made significant progress in studying digital media and immigrant identity. Research on Chinese immigrants’ identity in digital media highlights the critical role of digital platforms in shaping transnational cultural identities. For instance, Qiu (2020) specifically examines how Chinese digital diasporic media, such as WeChat, shapes Chinese immigrants’ identities, and emphasizes the role of digital media in maintaining connections with their homeland. Ju et al. (2021) demonstrate that WeChat serves as a bridge in the adaptation process of Chinese immigrants in Canada but may also create cultural bubbles that hinder connections with local communities. These studies reveal the complexity and diversity of digital media in shaping Chinese immigrants’ identities.

In the context of media studies on Mainland China and Hong Kong, Cao et al. (2014) highlight that, in situations involving cultural conflicts between Mainland Chinese and Hong Kong locals, both groups exhibit the third-person effect and hostile media effects (HMEs). They perceive media messages as biased against their own group, while most overseas Chinese (third persons) consider the same media reports to be objective and neutral. Similarly, Ong and Lin (2017) find that, on digital media platforms, the “othering” of Mainland Chinese and Hong Kong locals is distinct from typical descriptions of racism, as it represents an intra-ethnic form of racialized practice. Song and Wu (2018) further note that online discussion tools play a significant role in shaping public opinion in Mainland China and Hong Kong. They cite Weibo as an example, which provides a platform for broader participation and has emerged as an active agenda-setter and interpreter in conflicts.

Komito (2011) found that social media serves a dual role for immigrants: it provides a channel for maintaining connections with their countries of origin while also facilitates integration into local networks in the destination country. This enables immigrants to navigate transnational identities, balancing cultural affiliations with both their homeland and place of residence. In the light of the concept of “dual cultural belonging,” Brinkerhoff (2009) observed that first-generation immigrants tend to use digital platforms to preserve traditional culture, whereas second-generation immigrants leverage digital spaces to reconcile dual cultural identities. This phenomenon aligns with the perspective of “media generativity”: social media not only transmits cultural content but also reshapes immigrants’ everyday realities through interactive contexts. This suggests that digital spaces introduce new dimensions to immigrant cultural adaptation. Leurs (2020), based on research into refugees’ social media use, further argues that digital platforms enable marginalized immigrant groups to adapt to the information society, access public services, and integrate into mainstream society.

However, the identity negotiation of immigrants on social media is not without tensions. King and Wood (2010) note that technical designs, such as algorithmic recommendations and information homogenization, shape immigrants’ digital interaction experiences, potentially intensifying specific group consciousness. With the proliferation of ICTs (information and communication technologies), immigrants’ everyday practices exhibit characteristics of digital embeddedness. Traditional immigration studies often treat national borders as natural containers for identity, presuming that immigrants must choose their identity affiliation within a binary “country of origin–destination country” framework. Nedelcu (2012) argues that this perspective fails to account for the sustained transnational connections enabled by social media and instant messaging technologies in the digital era, as well as the resulting fluidity of identity. Matamoros-Fernández and Farkas (2021), Ponzanesi and Leurs (2022), and Oksanen et al. (2014) highlight that issues such as online hate speech and information cocoons exacerbate tensions between immigrants and mainstream society, undermining social cohesion. These findings urge researchers to critically assess both the opportunities and challenges of immigrant identity construction in the digital age. Lim and Pham (2016), in their study of Chinese immigrants’ digital communication practices, emphasize the importance of situating these practices within the socio-political context of the immigrants’ environment.

A review of existing literature reveals that digital media platforms introduce new dimensions to immigrants’ identity formation, providing online avenues for identity negotiation. Current research is largely limited to empirical studies exploring the relationship between social media and immigrant identity, with a primary focus on mainstream international platforms such as Facebook and Twitter. Examinations of regional forums and other digital spaces remain relatively scarce. Moreover, existing studies often rely on static methods such as content analysis and survey interviews, and lack in-depth analyses of the dynamic formation of identity in immigrants’ online interactions. There is an urgent need to integrate local contexts and innovate research perspectives and methodologies.

### 2.3 The issue of immigrant identity in the context of Hong Kong

The issue of immigrant integration in Hong Kong is characterized by its uniqueness. Under the “One Country, Two Systems” framework, differences in political, economic, and socio-cultural dimensions between Mainland China and Hong Kong create tensions for immigrant identity formation. Research indicates that Chinese immigrants face significant challenges in adapting to life in Hong Kong’s new social environment (Mo et al., 2006). These challenges include unacceptable housing conditions, unemployment, low income, discrimination, social isolation, and declines in social and economic status (Wong et al., 2011). Ngo and Li (2016) and Downes (2017) found that some Mainland new immigrants in Hong Kong encounter employment discrimination and interpersonal alienation, leading to a limited sense of identification with Hong Kong. Kwok-Bun and Peverelli (2013) further corroborate this, noting that earlier studies have revealed structural discrimination mechanisms against Mainland immigrants in Hong Kong, such as low-skilled immigrants being confined to informal economic sectors (e.g., catering and domestic services) and professional immigrants facing qualification recognition barriers. He et al. (2009) confirmed that Cantonese proficiency is a critical variable for social integration among Mainland new immigrants, with monolingual Mandarin speakers experiencing significantly higher unemployment rates than bilingual individuals. Other studies show that, compared to the local Hong Kong population, Mainland immigrants are less likely to secure employment after arriving in Hong Kong, and even when employed, their incomes are lower (Chiu et al., 2005; Chou, 2013; Zhang and Wu, 2011). Veg (2017), So and Ip (2019), and Downes (2017) point out that the rise of localist discourses in Hong Kong in recent years has posed identity challenges for Mainland immigrants. Peng (2016), through an analysis of identity negotiation among Mainland Chinese university students in Hong Kong, reveals their multifaceted transitions between identities such as Hong Konger, Mainlander, and global citizen. These studies highlight the complexity of immigrant identity negotiation within the Hong Kong context from various perspectives.

As digital platforms gain prominence in Hong Kong, the impact of immigrants’ online interactions on their identity construction has drawn increasing attention. Lee et al. (2021) note that during the Anti-Extradition Law Movement, forums like LIHKG became hubs for localist discourses, with some negative rhetoric targeting Mainland immigrants sparking controversy. Yuen and Chung (2018) provide contextual support through their theoretical analysis of localism, noting that localists often frame Mainland immigrants as “resource plunderers” or “cultural invaders,” a discourse that is easily activated during heightened political conflicts. Lee et al. (2015) highlight the strong public resentment in Hong Kong toward immigrants’ access to welfare benefits. Further argue that discrimination and exclusion faced by Mainland new immigrants from Hong Kong locals are closely tied to issues such as “healthcare, subsidized housing, educational resources, and social welfare.” However, there remains a lack of in-depth research on the identity negotiation of Mainland immigrants within such digital spaces.

In summary, immigrant identity is a critical area of cross-cultural research. Traditional theories have focused on immigrants’ offline cultural adaptation, but understanding of identity construction in digital environments remains underdeveloped. As digital life becomes the norm, social media has emerged as a vital arena for immigrants’ cross-cultural interactions and identity negotiation. Current research lacks comparative analyses of different types of digital platforms and in-depth explorations of the mechanisms of identity construction in immigrants’ online interactions. Within the Hong Kong context, understanding the identity practices of Mainland Chinese immigrants in local digital spaces represents a significant knowledge gap. In terms of research content, existing studies have largely overlooked the dynamic processes of identity negotiation, emotional expression, and group interactions among Mainland Chinese immigrants on Hong Kong’s digital media platforms, resulting in an insufficient comprehensive analytical framework for understanding immigrants’ digital lives. Most studies on immigration and social media focus on mainstream platforms in Western contexts, rendering the resulting “digital immigrant” theoretical frameworks less universally applicable and poorly suited to the unique “One Country, Two Systems” context of Mainland China and Hong Kong. In aspects of research methods, quantitative studies, speculative research, and case analyses dominate current investigations of the “digital immigrant” topic. These methods effectively identify core factors hindering new immigrants’ identity formation and offer practical guidance for their integration into local communities. However, they fall short of providing systematic analyses. Systematic analysis requires a more holistic consideration of the interplay among multiple factors—including technological, cultural, social, and economic dimensions—and necessitates long-term observation and tracking studies. By extending the time span and dimensions of research, a deeper understanding of the long-term impacts and adaptation processes of digital immigrants can be achieved. This study takes LIHKG as a case study, integrating grounded theory and online ethnography methods to deeply explore the mechanisms of identity negotiation in the digital lives of Mainland Chinese immigrants in Hong Kong. It aims to enrich theoretical understandings of the dynamic construction of immigrant identity and provide new perspectives for reflecting on immigrant integration practices in Hong Kong and broader contexts.

## 3 Research methodology

### 3.1 The grounded theory framework

This study adopts the grounded theory approach developed by Strauss and Corbin (1998), and takes following key aspects into considerations: (1) Grounded theory is highly suitable for exploratory research needs. In current studies on immigrant identity construction, particularly in the context of the digital era, there lacks well-developed and mature theoretical frameworks to interpret many phenomena comprehensively. Grounded theory excels in such scenarios, enabling researchers to explore new and insufficiently understood social phenomena, uncovering their underlying patterns and essence. (2) A core strength of this method lies in its advocacy for deriving theoretical frameworks directly from collected data, rather than relying on pre-established hypotheses for validation, as is common in traditional research. This data-to-theory generative pathway effectively mitigates the interference of preconceived notions on research outcomes, making it particularly adept at revealing localized characteristics and processes in immigrant identity construction. Through in-depth analysis of raw data, the study can precisely capture the unique modalities and internal logic of identity construction among immigrants within specific cultural, social, and regional contexts. (3) The systematic coding procedures of grounded theory provide a rigorous and standardized framework for qualitative data analysis. From open coding for initial concept extraction, to axial coding for establishing relationships between concepts, and finally to selective coding for identifying core categories and constructing theoretical models, each step follows a clear operational process and targeted objectives, ensuring the scientificity and reliability of the research process.

Throughout the research process, this study will employ a theoretical sampling strategy. This means that the research focus will not remain fixed but will be flexibly adjusted and refined based on the characteristics of the collected data and emerging clues or questions during the analysis. This iterative process continues until subsequent data collection no longer yields new information that significantly enriches or alters the existing theoretical concepts and models, reaching a state of theoretical saturation. This approach ensures that the constructed theory possesses robust explanatory power and universality.

### 3.2 Data collection strategies

#### 3.2.1 Data collection method

##### 3.2.1.1 Platform selection and keyword development

As of February 2025, according to Similarweb statistics, LIHKG ranks as the fourth most popular social media platform in Hong Kong, with a total of 24 million visits in February. In terms of PC traffic distribution by country/region, 87.44% of LIHKG’s traffic originates from Hong Kong. The platform’s primary audience is relatively young, with the largest age group of visitors being 25–34 years old. The user base is 64.56% male and 35.44% female. Additionally, LIHKG has a bounce rate (the percentage of visitors who leave after viewing only one page) of 25.08%, an average of 9.99 pages viewed per visit, and an average visit duration of 9 min and 49 s. Following thorough investigation and analysis, this study selected LIHKG as the primary source for data collection. The platform holds a dominant position in Hong Kong, with leading levels of local identification and user engagement. Both Hong Kong locals and Mainland Chinese immigrants in Hong Kong are highly active on the platform, providing rich and authentic primary data for studying immigrant identity construction. This unique user structure and vibrant atmosphere make LIHKG an ideal research field for observing interactions between Mainland immigrants and local residents. Concurrently, the study conducted in-depth interviews with 20 Mainland Chinese new immigrants in Hong Kong based on the research questions.

For keyword selection, we conducted a preliminary exploratory analysis of LIHKG discourse patterns related to Mainland Chinese immigrants. Through systematic observation of platform discussions over a three-month period (January–March 2024), we identified recurring terms and phrases used to reference or discuss Mainland Chinese immigrants. This process involved examining 200 randomly selected posts containing identity-related discussions to establish a comprehensive keyword landscape. Based on frequency analysis and semantic clustering, we developed a keyword taxonomy encompassing positive (“new Hong Kongers,” “Talent Admission Scheme”), neutral (“Mainlanders,” “new immigrants,” “One-way Permit,” “IANG visa,” “cross-border students”), and negative (“locusts,” “strong country people,” “Northerners”) identity markers (Kozinets, 2020). The final selection of keywords was validated through consultation with two Hong Kong media studies scholars familiar with local discourse patterns and refined based on pilot testing with a subset of 50 posts to ensure comprehensive coverage of immigrant-related discussions.

##### 3.2.1.2 Data scope and sampling considerations

The study sets the data collection period from 2018 to March 2025. During this timeframe, the focus is on collecting posts and replies on the platform that are closely related to Mainland immigrants using the validated keyword framework described above. To ensure data quality and relevance, we established specific filtering criteria: posts with total likes or dislikes exceeding 300 (representing the top 25th percentile of platform engagement based on our preliminary analysis) and at least 20 replies per post to ensure sufficient interaction depth for meaningful analysis.

We acknowledge that these engagement thresholds may introduce sampling bias toward more polarized or controversial content, as highly engaged posts on politically sensitive platforms often generate stronger reactions than typical discussions (Boyd, 2014). However, this approach was deliberately chosen to capture substantive identity negotiations where meaningful discourse occurs, rather than superficial mentions. Posts with minimal engagement often lack the depth of interaction necessary for grounded theory analysis of identity construction processes. To address potential bias, we supplemented our analysis with interview data to capture perspectives that may be underrepresented in high-engagement posts, including those who engage in self-censorship or lurking behaviors.

##### 3.2.1.3 Data Validation

To ensure the reliability of the collected data sources, this study employs multiple cross-validation methods. First, it leverages platform-specific features, such as the IP addresses displayed with user posts and language usage patterns, to preliminarily determine users’ identity attributes and regional origins. Second, it cross-references self-reported identity information provided by users in posts or replies with platform feature data. By combining these two approaches, the authenticity and reliability of user identities can be confidently established.

#### 3.2.2 The process of data collection

The data collection process of this study is divided into two main components: the collection of post data from the LIHKG platform and the collection of in-depth interview data from Mainland Chinese new immigrants. These two components complement each other, collectively forming the data foundation of the study.

##### 3.2.2.1 LIHKG platform post collection

The collection of posts employs a strategy combining keyword filtering with multiple criteria. First, the study identified over 20 keywords highly relevant to the research theme, including “Mainlanders,” “new Hong Kongers,” “Mainland new immigrants,” “Talent Admission Scheme,” “One-way Permit,” “IANG visa,” “Specialist Programme,” “cross-border students,” “going north,” “strong country people,” and “locusts.” These keywords encompass positive, neutral, and negative identity expressions to ensure data comprehensiveness. For instance, “Mainlanders” and “new immigrants” are neutral descriptors, “new Hong Kongers” reflects a positive integration identity, while “strong country people” and “locusts” represent derogatory labels, capturing a broad spectrum of perspectives. Second, the research team established stringent filtering criteria: (1) Timeframe: January 2018 to March 2025, covering periods of significant social changes in Hong Kong; (2) Interaction Heat: Total likes or dislikes exceeding 300 (a threshold determined based on the top 25th percentile of platform data distribution); (3) Interaction Depth: At least 20 replies per post to ensure sufficient depth for analysis. These criteria aim to select representative and information-rich discussion content.

Due to LIHKG’s technical characteristics and anti-scraping mechanisms, this study opted for manual data collection. The research team consisted of four researchers proficient in Cantonese and familiar with Hong Kong’s online culture, who collaboratively conducted manual searches, filtering, and data cleaning. Each researcher was responsible for collecting posts related to five keywords, using the platform’s built-in search function and recording qualified posts in chronological order. To ensure collection quality, all posts were cross-checked by a second researcher to confirm compliance with the filtering criteria. Ultimately, the team collected 800 posts, including original post content and all replies, forming a database of approximately 28,243 text units.

The collected content for each post includes: post title, publication date, anonymized user ID of the poster, post body, number of likes/dislikes, number of replies, full reply content with corresponding anonymized user IDs, and their respective likes/dislikes counts. All data were stored in a structured format, with an indexed system incorporating metadata to facilitate subsequent analysis.

##### 3.2.2.2 In-depth interviews with Mainland Chinese new immigrants

To validate and deepen understanding of online data, this study conducted in-depth interviews with 20 Mainland Chinese new immigrants. The participant recruitment process followed a systematic approach designed to ensure diversity and representativeness within our target demographic.

Recruitment Strategy and Process: Participants were recruited through multiple channels over a four-month period (September–December 2024). Initial recruitment occurred through Mainland student organizations at Hong Kong universities (*n* = 8 initial contacts), new immigrant social groups identified through community organizations (*n* = 12 initial contacts), and professional networks (*n* = 6 initial contacts). Snowball sampling was then employed, with each initial participant asked to refer up to two additional eligible individuals, resulting in 15 additional contacts. In total, 41 individuals expressed initial interest and completed screening questionnaires.

Selection Criteria and Exclusion Process: From the 41 potential participants, we applied systematic selection criteria: (1) Age Range: 22–30 years old, representing the primary demographic of contemporary immigrants; (2) Length of Residence in Hong Kong: 1–7 years, ensuring sufficient adaptation experiences; (3) Regional Diversity: Including individuals from Guangdong Province and non-Guangdong regions; (4) Occupational Diversity: Students, professionals, and entrepreneurs/freelancers; (5) LIHKG Usage Experience: All participants had experience browsing or engaging on LIHKG with minimum usage of 6 months and weekly platform visits.

Twenty-one participants met all criteria. One participant withdrew due to scheduling conflicts, resulting in our final sample of 20 participants. The final composition included individuals from Guangdong Province (6 participants) and non-Guangdong regions (14 participants), with occupational distribution of students (4), professionals (7), and entrepreneurs/freelancers (9). Platform usage intensity was assessed through self-reported metrics: average weekly usage ranged from 2 to 8 h, with 12 participants reporting daily browsing and 8 reporting 3–4 times weekly engagement. All participants demonstrated substantive platform familiarity through ability to discuss specific LIHKG cultural norms and recent discussions during screening interviews (Patton, 2015).

### 3.3 Data analysis procedures

Data analysis adheres to the three-stage coding procedure of grounded theory while addressing potential limitations inherent in this methodological approach:

Methodological Considerations and Limitations: We acknowledge that grounded theory’s strength in generating context-specific insights may potentially create tension between empirical specificity and theoretical abstraction (Charmaz, 2014). To address this concern, we maintained constant attention to the historical and sociopolitical context of Hong Kong-Mainland relations throughout our analysis, ensuring that theoretical categories remained grounded in specific temporal and spatial realities rather than becoming decontextualized abstractions. Our coding process explicitly incorporated contextual memos documenting how each emerging category related to broader Hong Kong political developments and specific platform dynamics.

Triangulation and Data Integration: The integration of post data and interview data employed a systematic convergence approach (Creswell and Plano Clark, 2017). During analysis, we identified both convergent patterns and divergent findings between online expressions and personal narratives. Notable convergences included consistent identity negotiation strategies across both data sources and similar emotional regulation patterns. Key divergences emerged in the expression of political opinions, with interview participants demonstrating more nuanced views than their online expressions suggested, reflecting the constraining effects of platform surveillance and audience considerations. These divergences were incorporated as analytical insights rather than methodological problems, illuminating the complex relationship between public and private identity work.

#### 3.3.1 Open coding

In the initial stage of data analysis, the study analyzed 800 posts to identify concepts related to identity expression, emotional expression, and group interactions, forming a preliminary coding framework. The coding process employed the constant comparison method as the core operational approach, continuously comparing new data with existing concepts. If the concepts embedded in new data were highly similar to existing ones, they were integrated into the corresponding coding categories; if new data revealed novel conceptual features, new coding categories were created to record them. Ultimately, the content from interviews with 20 participants was used to supplement the final theoretical model. Through comparison, induction, and supplementation, the resulting concepts were ensured to have high precision and broad inclusivity.

#### 3.3.2 Axial coding

The primary task at this stage was to deeply explore the intrinsic connections among initial concepts, thereby constructing organic relationships between categories and subcategories, ultimately forming the highly explanatory “conditions-actions-consequences” analytical paradigm. In practice, the analysis focused on the interrelationships among three key dimensions: identity negotiation strategies, emotional expression patterns, and interaction types.

#### 3.3.3 Selective coding

Based on axial coding, this stage involved identifying the core category, constructing a theoretical model, and validating the model’s explanatory power through theoretical sampling. This study established “identity negotiation on online platforms” as the core category, around which a highly logical and systematic theoretical framework was developed.

Data analysis was conducted by using the computer-aided qualitative data analysis software NVivo 15.0 for coding and theoretical memo recording, ensuring the systematicity and transparency of the analysis process.

### 3.4 Ethical considerations

This study received ethical approval from the Institutional Review Board of Tongji University (No: tjdxsr2024039). Ethical considerations encompassed both interview data collection and analysis of public platform content.

Interview Ethics: All interview participants provided written informed consent after receiving detailed information about study purposes, data usage, and their rights to withdraw. Interviews were conducted in participants’ preferred languages to ensure authentic expression. All audio recordings were encrypted and stored on password-protected devices, with transcripts anonymized immediately upon completion. Participants were assigned alphanumeric codes (e.g., P01, P02) to protect identity.

Platform Data Ethics: While LIHKG posts are publicly accessible, we implemented additional privacy protections given the sensitive political context surrounding Hong Kong-Mainland relations (Zimmer, 2018). All direct quotes from posts were paraphrased or substantially modified to prevent traceability while preserving analytical meaning. User handles and post metadata were completely anonymized. No screenshots or exact post reproductions were retained in research files. Given LIHKG’s anonymous nature, this approach ensures double anonymization while maintaining analytical validity.

## 4 Research context

### 4.1 Features of LIHKG platform

LIHKG, established in 2016, is a prominent locally-driven online forum in Hong Kong. According to a public opinion survey conducted by Apple Daily on July 1, 2019, 55% of respondents identified LIHKG as the most significant media platform (Apple Daily, 2019). LIHKG occupies a pivotal position in Hong Kong’s cyberspace due to its distinctive user culture and substantial social influence. The platform is characterized by the widespread use of local Hong Kong slang, internet jargon, and cultural symbols, fostering a unique community culture (Ting, 2024). As a local “meme field,” LIHKG emerged as a successor to the Hong Kong Golden Forum (HKGolden Forum), which had been popular since 2000, with users migrating to LIHKG following its inception in 2016 (Matthew, 2021). Since replacing HKGolden Forum, LIHKG has been implicated in discussions during significant events, such as the 2016 “Thunderbolt Plan” and the Mong Kok riots, where certain discourses were related with factual distortions and the incitement of antagonistic sentiments, seemingly aimed at misleading the public and generating social unrest. During the 2019 anti-extradition movement, the platform facilitated the dissemination of solidarity content through a user-driven upvote/downvote mechanism, effectively curbing divisive rhetoric (Leung et al., 2022). In the initial phase of the implementation of The Law of People’s Republic of China on Safeguarding National Security in the Hongkong Special Administrative Region in 2020, a notable number of anti-China and destabilizing posts denigrating the law appeared on the platform, though these were subsequently suppressed by platform moderation. The discursive environment across LIHKG’s various discussion boards has shown improvement during major events, including the refinement of Hong Kong’s electoral system in 2021 and the city’s integration into national development frameworks between 2022 and 2023. LIHKG users engage in discussions anonymously, yet their identity characteristics are manifested through linguistic choices and expressed viewpoints (Au et al., 2021; Erni and Zhang, 2022). Structured similarly to Reddit, LIHKG restricts posting privileges to registered users with email addresses registered in Hong Kong, a feature that significantly enhances information security for organizing offline activities and fosters collective strategizing (Albrecht et al., 2021; Lee and Tsui, 2020). This mechanism ensures the authenticity of users’ expressions of psychological states (Tsang, 2020). The platform’s anonymity enables users to seek out target communities based on personal privacy needs, facilitating interactions with peers who share similar experiences to gain emotional support, advice, social connections, and a sense of community belonging (Yu et al., 2023). By incorporating user voting to determine anonymity and “thread popularity” (Liang and Lee, 2021), LIHKG is perceived by users as a sanctuary for unrestrained political expression (Erni and Zhang, 2022). LIHKG encompasses a diversity of topics, including politics, society, culture, and everyday life, providing rich material for analyzing identity interactions across varied contexts (Leung et al., 2022). Particularly for political topics, users regard LIHKG as an broader space for creating and disseminating political content (Lee et al., 2021; Yeo, 2019). The platform operates through several key mechanisms: 1. Voting Mechanism: The upvote/downvote system establishes a community-driven evaluation framework, influencing users’ discursive strategies and identity expressions (Leung et al., 2022). 2. Topic Guidance Mechanism: LIHKG features distinct sections and topic categorizations, enabling users to engage in discussions aligned with their areas of interest. 3. Moderation Mechanism: The platform enforces specific moderation policies to filter out illicit, illegal, or inappropriate content, thereby maintaining the discussion environment’s order and ensuring functional interactions. 4. Prioritization Mechanism: LIHKG allows users to collectively prioritize significant content, with continuously updated threads (e.g., those receiving new replies) being elevated to the homepage (LIHKG’s “most popular” list), thereby gaining greater visibility (Liang and Lee, 2021).

### 4.2 Mainland immigrant groups

New immigrants from Mainland China to Hong Kong exhibit diverse profiles, including skilled migrants, family reunification migrants, student migrants, and other categories, each characterized by distinct socioeconomic backgrounds and adaptation needs. According to data from the Hong Kong Census and Statistics Department, between 2010 and 2023, over 800,000 Mainland immigrants arrived in Hong Kong through channels such as the One-Way Permit scheme and talent admission programs, accounting for approximately 11% of Hong Kong’s total population (Census and Statistics Department, 2025).

Under the framework of skilled migration policies, the Hong Kong government introduced the Top Talent Pass Scheme to attract high-income professionals and graduates from the world’s top 100 universities. From December 28, 2022, to September 2024, the scheme received over 100,000 applications, with 81,000 approved. Concurrently, the Global Talent Pass (which imposes specific requirements for academic qualifications and work experience) recorded 132,000 applications, with 21,000 approved. The Talent Plan (designed to meet the demand for specialized talent in local enterprises) received 43,000 applications, with 38,000 approved (Immigration Department, 2024).

Family reunification migration primarily pertains to the reunification of immediate family members, such as spousal or parent–child reunions, with eligibility contingent upon the applicant having an immediate family member who is a Hong Kong resident or permanent resident. In 2021, this category constituted 59% of total immigration to Hong Kong, with 22,000 family reunification migrants recorded in the first half of 2022. Student migration is tailored for non-local students who have obtained a bachelor’s degree or higher in Hong Kong, with eligibility for permanent residency after 7 years of residence post-graduation. From December 28, 2022 to September 2024, the student migration scheme received 47,000 applications, with 44,000 approved, of which 39,000 graduates remained in Hong Kong (Immigration Department, 2024). Throughout the protracted process of immigrant adaptation, new arrivals commonly encounter multiple challenges, including linguistic barriers, cultural disparities, social integration difficulties, and identity negotiation (Law and Lee, 2006).

### 4.3 Social and political background

Hong Kong and Mainland China share a common cultural and historical origin, yet their relationship has evolved dynamically through historical processes, with numerous pivotal events profoundly shaping bilateral ties and significantly influencing the identity formation of Mainland immigrants in Hong Kong (Ngo and Li, 2016). For instance, on June 29, 2003, the Mainland and Hong Kong Closer Economic Partnership Arrangement (CEPA) was signed. Through subsequent supplementary agreements, CEPA facilitated comprehensive advancements in goods trade, service trade, and investment liberalization, enabling Hong Kong’s financial and trade sectors to expand into Mainland markets (Ching et al., 2012; Chiu et al., 2005). This development spurred a significant influx of Mainland professionals to work and settle in Hong Kong, fostering increasingly multifaceted identity perceptions during their integration into Hong Kong society.

In the same year, on July 28, 2003, the Free Travel Policy was piloted in four Guangdong cities. As the policy expanded, the number of Mainland tourists visiting Hong Kong surged, substantially boosting the city’s tourism and retail sectors. However, this rapid increase in visitors also sparked tensions, with some Hong Kong residents developing antagonistic sentiments toward mainlanders (Wong et al., 2016). Against this backdrop, Mainland immigrants faced challenges in integrating into Hong Kong society, experiencing a diminished sense of belonging (Yang and Li, 2013).

From September 28 to December 15, 2014, the illegal “Occupy Central” movement disrupted Hong Kong’s political, economic, and social order, leading to a deteriorating investment climate (Lam, 2015). Mainland immigrants encountered heightened uncertainties in their life and investment in Hong Kong, which deepened their recognition of the importance of safeguarding national unity and reinforced their commitment to a dual identity (Bhatia, 2016; Leudar et al., 2004).

In 2019, the anti-extradition bill movement erupted, violent actions by Hong Kong’s opposition and radical factions severely undermined the city’s rule of law and prosperity. This turmoil inflicted significant economic damage and underscored the dangers posed by extremist forces, prompting Mainland immigrants to value the stability afforded by the “One Country, Two Systems” framework more deeply. While a group’s psychological sense of belonging can shape ideological orientations, external factors may also awaken consciousness to protect pre-existing ideological values (Chan, 2015; Stekelenburg and Klandermans, 2013). Consequently, Mainland immigrants strengthened their identification with the nation, resolutely opposing the separatist actions of Hong Kong’s extremist factions. In doing so, they clarified their values and identity positioning, contributing to the maintenance of Hong Kong-Mainland relations (Zhu and Chou, 2021).

These events witnessed the complex trajectory of Hong Kong-Mainland relations while driving the continuous reconfiguration of Mainland immigrants’ identity in Hong Kong. In the process, these immigrants have served as a vital bridge in fostering bilateral exchanges and cooperation. Collectively, these macro-level factors constitute the socio-political context for Mainland immigrants’ identity negotiation on the LIHKG platform, providing essential background for understanding their online interactions.

## 5 Data analysis and theoretical construction

### 5.1 Open coding: initial concept formation

Open coding, as the initial step in grounded theory, aims to deconstruct, examine, compare, conceptualize, and categorize raw data (Strauss and Corbin, 1998). In the open coding stage of this study, a line-by-line analysis of 800 posts from the LIHKG platform and interview data from 20 participants was conducted to identify preliminary concepts related to identity negotiation among Mainland Chinese immigrants. The coding process utilized the constant comparison method and was completed progressively across four stages: the first stage involved analyzing 200 posts to establish an initial conceptual framework; the second and third stages each analyzed 200 posts, iteratively refining and expanding the concepts; and the fourth stage analyzed an additional 200 posts to verify theoretical saturation. Concurrently, the interview data from 20 participants were integrated into the coding system to enhance the diversity and depth of the data.

Through the open coding process, this study identified 30 subcategories, which were organized according to the three core research questions: identity negotiation, emotional expression, and group interaction. Each subcategory emerged from recurring patterns in the raw data, reflecting the identity practices and modes of expression employed by Mainland Chinese immigrants on the LIHKG platform.

Each category comprises multiple conceptual encoding, which directly correspond to specific expressions within the raw data. For instance, the category of “ontological identity stability” encompasses three conceptual encoding: “essentialization of native identity,” “determinism of developmental trajectory,” and “persistence of cultural roots.” Each code is supported by corresponding verbatim statements from the data, such as, “Even after living in Hong Kong for so many years, nothing has really changed. No matter what they say, I’ve always felt that I am a mainlander” (essentialization of native identity) (see Tables 1–3).

**Table 1:** Categories related to identity negotiation.

Category	Conceptual encoding	Original statement
Ontological identity stability	Essentialization of native identity	“Even after living in Hong Kong for so many years, nothing has really changed. No matter what they say, I’ve always felt that I am a mainlander”
	Determinism of developmental trajectory	“I was born on the Chinese Mainland and grew up here. This fact cannot change my mindset.”
	Persistence of cultural roots	“I have maintained the values of people from the Chinese Mainland, but gradually I started to shift in the direction of the values in Hong Kong.”
Negotiation of hybrid identity	Identity duality awareness	“I’ve come to realize that I am a Hong Konger and at the same time a Chinese. I think it’s a process of the mixed nature of identity.”
	Transcending regional identity	“While I am Chinese, I also identify myself as a global citizen.”
	Identity capital complementarity	“In fact, having multiple or dual identities is quite advantageous. It’s not like what one might initially think, that one has to be a certain type of person, or that these two identities are in opposition. That’s really not the case.”
	Identity position fluidity	“And when I can switch between these identities freely, I think it’s quite typical.”
Dilemma of the identity marginalization	Dual othering experience	“I think it’s rather difficult for people who are not from Guangdong province to consider themselves as Hong Kong people.”
	Belongingness cognitive dissonance	“Especially when it comes to matters related to identity. Yeah, it’s rather negative because I feel that I still have not truly integrated into the local community.”
	Absence of collective belongingness	“It makes me feel as if I might be isolated. Maybe it’s because on the Chinese Mainland, it’s often about collective living or collective activities.”
Language as an identity marker	Linguistic-based discriminatory structure	“It’s just because I spoke in Mandarin. I feel that perhaps it was due to the fact that I was speaking Mandarin. Because I was chatting with my friends in Mandarin the whole time, the person taking our order, as I recall, rolled their eyes at me, and their service attitude was rather mediocre.”
	Language as a social stratification mechanism	“If you speak to him/her in Mandarin, he/she might be a bit slower in response. But if you speak to him/her in Cantonese, he/she will probably be quicker, and it can also reduce potential troubles.”
	Association between linguistic competence and regional exclusion	“That is to say, they would say that if you do not know the language, you should not come to Hong Kong. Anyway, they are just trying to discourage you and tell you to go back to the Chinese Mainland and not stay in Hong Kong.”
Strategies for linguistic capital accumulation	Progressive acquisition of linguistic capital	“When I first came here, I did not really know how to speak Cantonese. But as I spent more time living here, I gradually learned to understand it and became more willing to speak it.”
	Active linguistic capital investment	“After coming to Hong Kong, I would try to speak Cantonese with the people around me.”
	Context-oriented nature of linguistic capital	“When going out, in order to communicate more conveniently, I may choose to speak Cantonese.”
Multimodality of language practices	Context-driven code-switching	“For people from the Chinese Mainland, I will use simplified Chinese characters and Mandarin as the form of language expression when communicating with them, and I will not use so many internet meme.”
	Platform-specific language acquisition	“I choose to use Cantonese or English to post messages, and mainly use Cantonese as the language when making posts.”
	English as neutral linguistic capital	“When I talk to people in Hong Kong, for example, I use traditional Chinese characters along with Cantonese-specific characters to chat.”
Internalization of behavioral norms	Public transportation cultural adaptation	“When taking the MTR, before reaching the turnstile, I will quickly take out my Octopus card and try my best not to cause trouble for the people behind me. That is, I do not want to make them wait even for an extra second, because if they do have to wait for one more second, some Hong Kong people may get quite unhappy.”
	Adherence to orderly norms	“It includes giving up seats on the bus, and also when taking the elevator, people line up row by row.”
	Internalization of regulatory awareness	“For example, in Qingdao, one can smoke on the streets in some areas. However, in Hong Kong, smoking is prohibited. I was even fined for smoking before because I did not know the rule.”
Reshaping of social habitus	Adjustment of private discourse patterns	“Generally speaking, when having private conversations on the street, they tend to speak a little more softly.”
	Adaptation to transactional interaction patterns	“For example, when having a meal, they usually go Dutch.”
	Differences in service interaction patterns	“For instance, Hong Kong people may attach great importance to efficiency and do things very quickly, accurately and resolutely. And sometimes, it may seem that they are not that polite. On the other hand, people on the Chinese Mainland may have been influenced by the concept that “customers are gods” in their daily lives.”
Acquisition of institutional capital	Organized identity engagement	“I have participated in the Student Union and actively sought cooperation with local merchants in Hong Kong.”
	Peer network construction	“I’m quite familiar with several of them. Then they invited me to join them and we had fun together.”
	Performative practices on social media	“Regarding the interactions at school, we have academic cooperation within the school. Also, we organize activities like making friends together. In this process, we may post some group photos on social media platforms or leave messages for each other and so on.”

**Table 2:** Categories related to emotion expression.

Category	Conceptual encoding	Original statement
Stereotyped experiences	Encounters with linguistic stereotypes	“There is indeed some discrimination based on their stereotypes. For example, you yourself might use certain words or expressions in a way that reflects this.”
	Presumptive identity labels	“They have such a very strong stereotype, and it will give you a presumption.”
	Negative Framing of occupational identity	“Most people from the Chinese Mainland may be relatively poor. And if you are from the Chinese Mainland, you cannot afford to buy clothes.”
Platform space exclusion	Patterns of discursive exclusion	“I was using Mandarin to post messages. When I first entered this field to look for a job, I would use Mandarin and try to find people who also came here from the Chinese Mainland and were looking for a job, so as to communicate with them. However, the usual result was that many local Hong Kong users would criticize me under the posts.”
	Mechanisms of identity categorization and differentiation	“On this platform, it feels like it’s not guided by exchanges of stances or viewpoints. Sometimes, it may be more about making distinctions about you not based on your ideology but rather based on your identity, specifically your place of birth.”
	Self-censorship of expression	“I will not make too many comments on things that I do not understand.”
Internalization and resistance to exclusion	Silence as a coping strategy	“They think that you are brainwashed. When such remarks are made, I basically stay silent and will not reply.”
	Self-restriction of expressed content	“There were such situations before. But due to being attacked several times, or those intentional or unintentional verbal provocations, I rarely post my own emotional content anymore.”
	Emotional defense mechanisms	“To be honest, I’m a very vulnerable person. In such situations, my approach is that if it does not affect me directly, I will not take any action. But I will quietly set my ID to be visible only to those who follow me mutually. I guess this is a way of protecting myself.”
Conflict avoidance techniques	Avoidance of political discourse	“I will not communicate with them about my own political stance either. Even if they bring up this topic with me on their own initiative, I still will not engage in such a conversation.”
	Maintaining surface neutrality	“I just feel that I should remain neutral. Even if others ask me about it, I will also say that I am neutral and will not get involved in this matter.”
	Minimization of platform engagement	“Most of the time, I try to avoid such situations. Sometimes, I just stay silent and lurk around.”
Identity performance strategies	Concealment of identity markers	“They will also hold some discrimination or prejudice against you. That’s why I will try my best to avoid revealing my identity or expressing my identity.”
	Adoption of alternative linguistic symbols	“If it’s on Instagram or LIHKG, I generally post in English.”
	Intentional avoidance of identity markers	“I will not specifically say on the media that I’m a Hong Konger of China or anything like that. I’m just trying to avoid such sensitive topics because I’m afraid they will not like to hear it.”
Rationalized coping strategies	Conditional expression of opinions	“If the other party talks in a reasonable way, that is, they communicate with me in a relatively normal, kind and friendly manner, then I am able to objectively express my own views.”
	evidence-oriented argumentation approach	“It’s mainly about communication and reasoning. That is, one might present some news, research findings or evidence to express one’s own opinions, instead of just venting one’s emotions.”
	Standardization of discursive techniques	“When arguing for a certain matter, I will be very serious and adopt the approach similar to that on Zhihu. For example, I will introduce evidence, add some links to the original texts, and also present data to support the viewpoints I put forward.”
Tendency toward homogeneous interaction	Preferential interaction with homogeneous groups	“There are also some people on LIHKG who, like me, are new arrivals in Hong Kong from the Chinese Mainland. They also post messages, and I will also post messages to communicate with each other. Maybe I will describe my own experiences in Hong Kong, including aspects such as study, entertainment, and social life. They will also show their approval for these, and I will do the same.”
	Connection with individuals of shared experiences	“Relatively speaking, maybe if you only communicate with those local people, some of them will be rather hostile towards you, making it difficult for you to integrate. However, if you communicate with people who have similar experiences to yours, this problem will not arise.”
	Creation of safe social spaces	“After communicating with them on that platform, I successfully met them offline and made friends. I think it’s great to communicate and associate with people who are like-minded in this way. “
Cross-group collaboration strategies	Functional mutual support relationships	“I will look for some group members on it. We have different courses. For some courses, forming teams is required. Some local people will post invitations for teaming up on it. Previously, a pair of local people invited me to join their team.”
	reciprocal communication	“Our strategy is to exchange. I’ll give them my formula, and they’ll give me theirs.”
	Co-creation effect of cultural outcomes	“When you create a wonderful cultural product together, that’s probably the moment when their sense of identification towards you and the so-called sense of belonging are at their strongest.”
Exchange of cultural capital	Practice of the principle of cultural conformity	“Since I have chosen to come to this place, I think I still need to respect the local culture.”
	Expression of reciprocal respect	“It’s about respecting each other’s cultures.”
	Strategic adjustment of communication style	“Just try to speak in a kind and normal tone, and then the other party will be more likely to accept what you say.”
Platform structural bias	LIHKG as an exclusionary field	“On LIHKG, it is mainly used by local people in Hong Kong. I rarely, rarely see people posting articles in simplified Chinese characters or in written language.”
	Evolution of platform discursive climate	“A few years ago, when I browsed LIHKG, I felt that there were more negative news about the Chinese Mainland. But now, I think there are more and more positive news about it.”
	Polarization of platform user ideologies	“Because the people on this platform are originally more radical. I think they are more nativist.”
Digital anonymity effect	Disinhibition behavior patterns	“Then on the LIHKG platform, if you act as a keyboard warrior, that is, once you connect the network cable and then when you unplug it, no one can see you. Then you will surely say whatever you want. I think this is quite obvious in Hong Kong.”
	Inconsistency between online and offline behaviors	“Many Hong Kong people, as you know, may not say much in daily life, but on the Internet, when they are online, they speak irresponsibly.”
	Symbolized exclusion due to simplified characters	“In fact, I think that on that platform, many people do not even care about your viewpoints sometimes. As long as they see that you are someone who writes in simplified Chinese characters, they will start to launch attacks right away.”
Influence of socio-political context	Polarization during social movements	“For example, during the so-called “anti-extradition bill” incident in 2019, they were divided into the “blue camp” and the “yellow camp.” They might often discuss such sensitive topics on LIHKG. Many people from the “yellow camp” would attack those from the “blue camp.””
	Group antagonism during the pandemic period	“During the COVID-19 pandemic, it was also particularly obvious. And later on, things gradually became more peaceful.”
	Identity Safety threats during politically sensitive periods	“I think during the riots in 2019, they would conduct “doxxing” on some relatively well-known Mainland students.”

**Table 3:** Categories related to group interaction.

Category	Original statement	Conceptual encoding
Accumulation of linguistic capital	Active acquisition of linguistic capital	**“**Although I cannot speak Cantonese very well, I try my best to communicate with them in Cantonese.**”**
	Socialized language acquisition	**“**I’m also gradually learning from my peers and observing the way they live.**”**
	Utilization of linguistic symbol systems	**“**I will also use the language of Hong Kong people, that is, traditional Chinese characters and Cantonese colloquial expressions to communicate.**”**
Conversion of capital within the education system	Challenges in the cross-border conversion of academic capital	**“**In Guangzhou, dealing with English is not that difficult. When I first arrived here, I simply could not keep up and had a really tough time studying it.**”**
	Reconstruction of learning values	**“**One thing I was really not used to when I first came to Hong Kong was that they did not care much about academic grades.**”**
	Investment in oral expression capabilities	**“**I ever did presentation, and answered questions in Cantoness, which has made my accent less pronounced.**”**
Cultivation of social capital	Adaptation to social rhythms	**“**Later on, I gradually got used to it. It’s just that this society has a relatively fast pace, and there is also a relatively high level of hostility and irritability.**”**
	Transformation of media usage patterns	**“**I think it’s the bandwagon effect. When people around me all started using these apps and got exposed to this mainstream social media culture.**”**
	Internalization of autonomy values	**“**I still decided to try to fit into this kind of life. Maybe I should get myself more accustomed to doing things alone or completing tasks independently, instead of being overly dependent on others.**”**
Shared cultural space	Cultural consumption consensus	**“**Regarding the visit of Lionel Messi to Hong Kong for a football match and the release of the Black Myth Wukong last year, they have achieved a form of group cooperation. Or at least, everyone has reached a consensus and shares the same views and opinions.**”**
	Survivalism consensus	**“**In fact, in real life, the Hong Kong people are also tired from constantly rushing around. And not many of them will deliberately bother to argue about whether you are from Hong Kong or the Chinese Mainland.**”**
	Cultural affinity cognition	**“**I think the people in Guangdong have a fairly high degree of cultural recognition of Hong Kong.**”**
Cross-boundary professional interaction	Neutral space for professional interaction	**“**During the process of my cooperation with him, there were not any internal conflicts that I had in mind. On the contrary, we had harmonious exchanges and discussions with each other.**”**
	Reciprocal teaching relationship	**“**He patiently explained my statements and provided me with the kind of replies that I had hoped to obtain in my ideal situation.**”**
	Empathetic interaction transcending identity	**“**The classmates at that time were really nice. They did not make fun of me even though I completely did not understand and could not keep up at all.**”**
Cross-perspective thinking	Perspective-taking ability	**“**The reason is that none of them stood in the other party’s position to think.**”**
	Dialectical judgment ability	**“**Actually, I’m not someone who particularly likes to post things online. For example, if I see a post where there is a war of words between Mainland people and Hong Kong people in the comments, I might take a look. Whichever side, whether it’s the Mainland people or the Hong Kong people, makes sense, I will give them a thumbs-up. If the Mainland people make sense, I’ll give them a thumbs-up, and if the Hong Kong people make sense, I’ll do the same for them. That is, I stand by the truth, not by my kin. I think this is very important. People should have a dialectical way of thinking and not let their stance determine their way of thinking.**”**
	Efforts toward depoliticized communication	**“**I think it’s most important to look at a problem calmly and not launch personal attacks just because of someone’s identity.**”**
Dilemma of mutual trust	Differences in trust systems	**“**I think trust is a very difficult problem to solve.**”**
	Incompatibility of communication modes	**“**He will only resort to verbal abuse or violent behavior. He will not listen to you carefully, nor will he talk to you properly.**”**
	Hyper-politicization of social issues	**“**When you talk with the professor and the topic comes to political democracy, it seems that everyone gets really excited, I think. It’s really difficult to communicate because they have their own very strong opinions and expressions, and the way I usually deal with it is just to keep silent.**”**
Ideological polarization	Solidification of group stereotypes	**“**For many Hong Kong people, their impression of the Mainland still remains at the level of mindless criticism.**”**
	Differences in early socialization	**“**The difficulty, as I see it, is that people’s ideological concepts are instilled from a young age, and there is also the issue of cultural identity.**”**
	Deeply entrenched political stances	**“**I think in 2019, during that riot, they would conduct doxxing on some Mainland students who were relatively well-known.**”**
Framework of resource allocation conflict	Perception of resource competition	**“**They think that we are competing with them and that we are taking away some of the resources that they originally should have had.**”**
	Zero-sum view of economic opportunities	**“**Mainlanders are believed to have affected their employment and also their welfare.**”**
	Divergence in political and economic systems	**“**Hong Kong practices capitalism, while the Chinese Mainland adheres to socialism. The Chinese Mainland places more emphasis on some collective activities, whereas Hong Kong is relatively more independent and autonomous in certain aspects.**”**

### 5.2 Axial /selective coding: category relationship construction and core theory development

Axial coding seeks to reorganize concepts derived from the open coding phase by employing the “conditions-actions-consequences” analytical paradigm, thereby establishing systematic connections between categories and subcategories (Strauss and Corbin, 1998). In this phase, the study integrated the 30 subcategories into nine main categories, which were further synthesized into three core categories, constructing a hierarchically structured categorical system as outlined below (see Table 4).

**Table 4:** Results of axial coding.

Core category	Main category	Category
Core category one: dialectical identity reconstruction		
Dialectical identity reconstruction	Ontological identity tension	Ontological identity stability
		Negotiation of hybrid identity
		Dilemma of the identity marginalization
	Linguistic symbolic capital	Language as an identity marker
		Strategies for linguistic capital accumulation
		Multimodality of language practices
	Reconstruction of social participation	Internalization of behavioral norms
		Reshaping of social habitus
		Acquisition of institutional capital
Core category two: emotion-right negotiation		
Emotion-right negotiation	Exclusion structure and coping strategies	Stereotyped experiences
		Platform space exclusion
		Internalization and resistance to exclusion
	Emotional labor strategies	Conflict avoidance techniques
		Identity performance strategies
		Rationalized coping strategies
	Group interaction strategies	Tendency toward homogeneous interaction
		Cross-group collaboration strategies
		Exchange of cultural capital
	Digital field rights topography	Platform Structural Bias
		Digital anonymity effect
		Influence of socio-political context
Core category three: transcultural capital conversion		
Transcultural capital conversion	Cultural capital investment	Accumulation of linguistic capital
		Conversion of capital within the education system
		Cultivation of social capital
	Construction of intercultural dialogue	Shared cultural space
		Cross-boundary professional interaction
		Cross-perspective thinking
	Structural integration barriers	Dilemma of mutual trust
		Ideological polarization
		Framework of resource allocation conflict

This categorical framework elucidates the multifaceted dimensions of identity negotiation among Mainland Chinese immigrants:

The first core category, “Dialectical Identity Reconstruction,” focuses on the internal mechanisms of identity formation. It encompasses ontological identity tensions, the pivotal role of language as a symbolic marker of identity, and the concrete practices realized through social participation.

The second core category, “Emotion-Right Negotiation,” centers on emotional management and interaction strategies within contexts of power asymmetry. It reflects coping mechanisms in response to exclusion, strategic practices of emotional labor, interaction patterns across diverse groups, and the distinctive power structures inherent to digital platforms.

The third core category, “Transcultural Capital Conversion,” addresses the process of capital transformation in cultural adaptation. It highlights proactive practices of capital investment, efforts to construct cross-cultural dialogues, and the persistent impact of structural barriers.

Through this hierarchical categorical system, the study not only delineates the interconnections among categories but also illuminates the systemic relationships between conditions, actions, and consequences in the identity negotiation process. This framework lays a robust foundation for the development of an integrated theoretical model.

### 5.3 Selective coding: core theory construction

**Figure 1:**
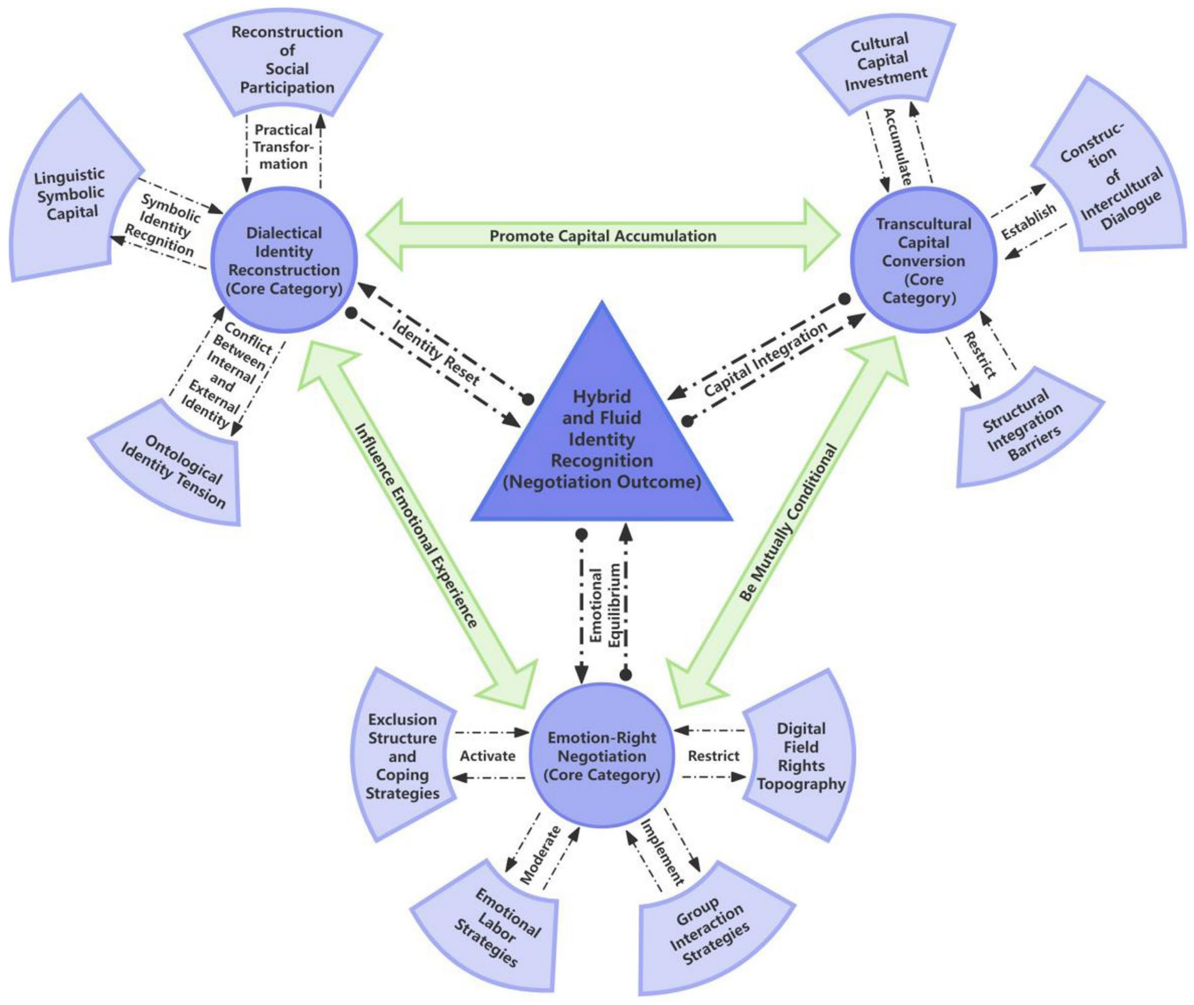
Theoretical model of identity negotiation for Mainland Chinese immigrants on the LIHKG platform.

Selective coding represents the final stage of theory construction, aiming to integrate all major concepts around core categories to form a coherent explanatory framework (Strauss and Corbin, 1998). Through iterative comparison and refinement, this study developed a theoretical model centered on “identity negotiation on digital platforms.” This model illustrates how Mainland Chinese immigrants navigate identity adaptation, express emotions, and engage in social interactions on the LIHKG platform (see Figure 1).

The storyline is as follows: Mainland Chinese immigrants, within the socio-cultural context of Hong Kong and particularly through interactions on online platforms such as LIHKG, exhibit complex and multifaceted dynamics of identity negotiation. The study reveals that this process revolves around three core categories: dialectical identity reconstruction, emotion-right negotiation, and transcultural capital conversion.

In the process of dialectical identity reconstruction, new immigrants experience ontological identity tensions, simultaneously maintaining an essentialist identity rooted in their native experiences while attempting to develop hybrid identities to cope with their new environment. However, they frequently encounter dilemmas associated with liminal identity spaces. These immigrants actively invest in linguistic symbolic capital, perceiving language proficiency as both an identity marker and a tool for social mobility. Through strategies of linguistic capital accumulation and multimodal language practices, they seek to reconcile identity contradictions. Concurrently, through social participation, they internalize local behavioral norms, reshape social habits, and acquire institutional capital, thereby achieve material basis through identity transformation.

The dimension of emotion-right negotiation elucidates the strategic responses of new immigrants to right asymmetries. They go through exclusionary structures and coping processes, encountering stereotyping and spatial exclusion on the platform, and develop internalized and resistant mechanisms such as silence and self-restriction. Through emotional labor strategies, they employ conflict avoidance techniques, identity performance, and rationalized coping mechanisms to manage emotional expressions and mitigate exclusion. Additionally, they cultivate group interaction strategies, gravitating toward homogenous interactions to foster a sense of security while also pursuing cross-group collaboration and cultural capital exchange. These interactions are restricted by the digital field rights topography, manifested through platform structural biases, the digital anonymity effects, and the influence of socio-political contexts.

Meanwhile, new immigrants actively engage in transcultural capital conversion. Through cultural capital investment, they acquire linguistic and educational capital and foster social capital. They strive to construct cross-cultural dialogues by seeking shared cultural spaces, developing transboundary professional interactions, and cultivating cross-perspectival thinking. Nevertheless, they continue to confront structural barriers for integration, including Dilemma of Mutual Trust, ideological polarization, and Framework of Resource Allocation Conflict.

This study reveals that the identity negotiation of Mainland Chinese immigrants is a dynamic dialectical process. They are both subjects of cultural right structures and agentic actors. Through the accumulation of linguistic symbolic capital, the deployment of emotional labor strategies, the learning of social field rules, and the construction of cross-cultural dialogue spaces, they constantly seek balance between “Hong Konger” and “Mainlander” identities. This process fosters the development of hybrid and fluid identities. By employing selective interactions, emotional regulation, and capital transformation strategies, immigrants negotiate identity, express emotional experiences, and establish interaction patterns with diverse groups on the LIHKG platform.

### 5.4 Conditions-actions-consequences analysis of the theoretical model

The theoretical model constructed in this study can be further elucidated through the “Conditions-Actions-Consequences” analytical paradigm:

Conditions: The identity negotiation of Mainland Chinese immigrants on the LIHKG platform is influenced by multiple conditions, including: At the individual level: native identity background and cultural capital; At the interactional level: platform exclusionary structures and digital anonymity effects; At the macro level: socio-political context and Framework of Resource Allocation Conflict.

Actions: In response to these conditions, new immigrants adopt a series of strategic actions, including, Linguistic capital accumulation, Internalization of social norms, Emotional labor practices, Selective interaction participation and Cross-cultural dialogue construction. These actions reflect the initiative of new immigrants while being constrained by structural limitations.

Consequences: These negotiation practices produce diverse outcomes, including: Fluidity and hybridity of identity, Constraint and release of emotional expression, Accumulation and transformation of social capital, Promotion and obstruction of cross-cultural understanding.

The core insight of the theoretical model lies in the following: the identity negotiation of Mainland Chinese immigrants is not a one way process of assimilation or resistance but a dynamic dialectical practice within unequal right structures. Through strategic actions, they seek a balance between maintaining native identity and integrating into the new environment, demonstrating initiative and resilience in the face of exclusion and prejudice. Meanwhile, the digital platform both provides a space for identity experimentation and reproduces the rights inequalities of the real world, forming a unique field for identity negotiation. This theoretical model not only enriches the theoretical perspective of migration identity studies but also provides a new analytical framework for understanding the complexity of cross-cultural communication in the digital era.

## 6 Model elaboration

### 6.1 Identity negotiation model in digital space

Through the analysis of 800 posts on the LIHKG platform and in-depth interviews with 20 participants, this study identifies four typical identity negotiation patterns exhibited by Mainland Chinese immigrants in Hong Kong’s digital space, which are discussed as below. These patterns are directly derived from the key categories identified in Chapter Five through open coding and axial coding, specifically “Negotiation of Hybrid Identity,” “Identity Performance Strategies,” “Rationalized Coping Strategies,” and “Conflict Avoidance Techniques.”

The integrative negotiation pattern is characterized by active adjustment of language use and adoption of local cultural symbols, while consciously integrating into the local cultural environment while retaining certain original cultural traits. In interviews, multiple participants expressed a proactive willingness to learn Cantonese and use traditional Chinese characters, as one participant stated: “I will also use the language of Hong Kong people, that is, traditional Chinese characters and Cantonese colloquial expressions to communicate” This pattern essentially transcends the simplistic assimilation strategy outlined in Berry’s (2005) acculturation theory, instead exhibiting a more dynamic and complex process of cultural integration. Particularly on online platforms like LIHKG, the acquisition of linguistic symbolic capital becomes a core component of new immigrants’ integration strategies, resonating with He et al.’s (2009) findings on language proficiency as a critical variable in social integration.

The confrontational negotiation pattern is manifested in the emphasis on original identity traits and proactive challenges to negative stereotypes about Mainland immigrants. This pattern frequently appears in post analysis, characterized by targeted rebuttals of discriminatory remarks and assertions of Mainland contributions to Hong Kong’s development. As one participant articulated their coping strategy: “It’s mainly about communication and reasoning. That is, one might present some news, research findings or evidence to express one’s own opinions, instead of just venting one’s emotions.” This pattern aligns closely with the social competition strategy in Turner and Tajfel’s (1986) social identity theory, reflecting the efforts of marginalized groups to challenge existing intergroup rights structures through collective action. The study finds that this pattern is particularly prevalent in discussions involving resource allocation and political issues, which corroborates Lee and Chou’s (2015) research on the tensions between Mainland new immigrants and local residents in competition for healthcare, housing, and educational resources.

The collaborative negotiation pattern, while acknowledging differences, actively seeks common ground and strives to construct a “third space” identity. In the analysis of posts and interviews, some new immigrants attempt to transcend binary oppositions by adopting hybrid identity labels such as “new Hong Konger” or emphasizing the advantages of cross-cultural competence. As one participant expressed: “In fact, having multiple or dual identities is quite advantageous. It’s not like what one might initially think, that one has to be a certain type of person, or that these two identities are in opposition. That’s really not the case.” This pattern resonates with Peng’s (2016) findings on Mainland Chinese students in Hong Kong navigating multiple identities as Hong Kongers, Mainlanders, and global citizens, reflecting Bhabha’s (1994) concept of the third space in cultural hybridity theory. Interview data indicate that this pattern is more prevalent in non-political topics and professional domain interactions, providing a potential space for identity negotiation.

The avoidance negotiation pattern is characterized by strategically reducing identity salience, by emphasizing individual traits over group identity. This pattern is particularly prevalent on the LIHKG platform, manifested in strategies such as avoiding sensitive topics, using neutral language, or employing English. As one participant noted: ““If it’s on Instagram or LIHKG, I generally post in English,” and “Most of the time, I try to avoid such situations. Sometimes, I just stay silent and lurk around.” These strategies align closely with Goffman’s (1963) stigma management strategies, reflecting the central role of information control in mitigating identity conflicts. This pattern is more pronounced in the platform’s anonymous environment, consistent with Erni and Zhang’s (2022) findings on LIHKG as a “safe haven for wild political expression,” yet it also underscores the self-protection mechanisms employed by new immigrants in this context.

It is noteworthy that these four negotiation patterns are neither static nor mutually exclusive but exhibit context-dependency and strategic fluidity. Research data indicate that the same immigrant may dynamically shift between different negotiation strategies based on factors such as the nature of the topic, the interactional counterpart, and the platform’s atmosphere, displaying marked variations across different sections or thematic discussions. This aligns with Leung et al.’s (2022) findings on the diverse topical spaces of the LIHKG platform. Such pattern shifts underscore the high flexibility and strategic nature of identity construction in digital spaces, challenging the assumptions of static adaptation types in traditional acculturation theories and resonating with Bhatia and Ram’s (2009) perspective on the tensions and struggles in the process of identity construction.

### 6.2 Emotion expression and identity construction

This study, through the analysis of posts and interview data from the LIHKG platform, reveals the central role of emotional expression in the identity negotiation process, directly linked to the categories identified in Chapter Five, including “Internalization and Resistance to Exclusion,” “Emotional Defense Mechanisms,” and “Shared Cultural Space.”

The dual emotional structure is a prominent feature of emotional expression among Mainland Chinese immigrants, characterized by the simultaneous expression of nostalgia for their original culture and curiosity/anxiety toward the new environment, resulting in a complex emotional state. In post analysis, this emotional duality is often presented through narrative contrasts. As one participant articulated: “I have maintained the values of people from the Chinese Mainland, but gradually I started to shift in the direction of the values in Hong Kong.” This emotional complexity resonates with Svašek’s (2010) description of the “bidirectional” nature of migrants’ emotional experiences and aligns with Mo et al.’s (2006) research on the emotional challenges faced by Chinese immigrants adapting to life in Hong Kong. Specifically, it manifests as new immigrants simultaneously expressing attachment to their original lifestyle and efforts to adapt to the new social rules of Hong Kong society on the platform. As one participant noted: “Later on, I gradually got used to it. It’s just that this society has a relatively fast pace, and there is also a relatively high level of hostility and irritability.”

Emotion as a resource in identity negotiation is manifested in immigrants strategically regulating emotional expression to garner social support and establish group connections. Data analysis reveals that moderately expressing vulnerability and adaptation difficulties can elicit empathy and support, whereas excessive negative emotional expression may lead to exclusion. This strategic deployment of emotional resources reflects immigrants’ initiative within unequal rights environments. As one participant described: “Active Linguistic Capital Investment: ‘After coming to Hong Kong, I would try to speak Cantonese with the people around me.’” This phenomenon aligns with Yu et al.’s (2023) research on the emotional exchange functions of the LIHKG forum, particularly the role of the platform’s anonymity in facilitating authentic emotional expression.

The formation of emotional communities is a significant outcome of platform interactions, prominently manifested in the categories of “Tendency Toward Homogeneous Interaction” and “Creation of Safe Social Spaces.” Through shared emotional experiences, specific emotional communities are formed on the platform, providing new immigrants with emotional support and a sense of belonging. As one participant stated: “After communicating with them on that platform, I successfully met them offline and made friends. I think it’s great to communicate and associate with people who are like-minded in this way. “This phenomenon resonates with Appadurai’s (1996) concept of “Emotional Geography,” referring to communities formed through emotional connections that transcend geographical spaces. It also corroborates McGregor and Siegel’s (2013) findings on social media as a transnational social space for the emotional support of immigrants.

The development of emotion regulation strategies is a critical component in immigrants’ responses to negative emotions, directly corresponding to categories such as “Emotional Defense Mechanisms” and “Silence as a Coping Strategy.” Interviewees exhibited a diverse array of coping mechanisms, including cognitive reappraisal: “In fact, in real life, the Hong Kong people are also tired from constantly rushing around. And not many of them will deliberately bother to argue about whether you are from Hong Kong or the Chinese Mainland”; seeking social support: “Relatively speaking, maybe if you only communicate with those local people, some of them will be rather hostile towards you, making it difficult for you to integrate. However, if you communicate with people who have similar experiences to yours, this problem will not arise.”; and emotional self-protection: “To be honest, I’m a very vulnerable person. In such situations, my approach is that if it does not affect me directly, I will not take any action. But I will quietly set my ID to be visible only to those who follow me mutually. I guess this is a way of protecting myself.” These strategies resonate with Gross's (2015) emotion regulation theory, yet they manifest distinct patterns within the context of cultural adaptation.

The study further revealed that transformations in emotional expression patterns often foreshadow identity transitions, progressing from initial feelings of alienation and anxiety toward a sense of belonging and identification, thereby constituting the emotional trajectory of the adaptation process. This finding challenges the traditional neglect of emotional factors in migration studies, underscoring the pivotal role of emotions as a mediating mechanism in cognition, social interaction, and identity construction.

### 6.3 Interaction patterns and social integration

This study systematically analyzed the interaction patterns between Mainland Chinese immigrants and local residents on the LIHKG platform, which were derived from “Cross-group Collaborations Strategies,” “Platform Space Exclusion,” and “Cross-boundary Professional Interactions” outlined in Chapter 5. The analysis revealed the following key findings:

Topic-Differentiated Interaction emerged as the most prominent interaction characteristic. Post analysis indicated that group boundaries were more pronounced in politically sensitive topics, with conflict and confrontation occurring more frequently. In contrast, interactions in domains such as daily life and career development exhibited a more inclusive atmosphere, with cross-group collaboration being more prevalent. As one interviewee described: “I will look for some group members on it. We have different courses. For some courses, forming teams is required. Some local people will post invitations for teaming up on it. Previously, a pair of local people invited me to join their team.” This finding challenges the oversimplified view of intergroup relations as unidimensional, highlighting the differential impact of interaction contexts. It aligns with Song and Wu’s (2018) research on the issue-specific nature of Hong Kong-Mainland online discussions.

Boundary Negotiation Mechanisms operated through subtle practices such as language use and cultural symbol referencing. Immigrants strategically adopted local vernacular and responded to local cultural memes to navigate or reconfigure group boundaries. Several interviewees highlighted language-switching strategies: “For people from the Chinese Mainland, I will use simplified Chinese characters and Mandarin as the form of language expression when communicating with them, and I will not use so many internet meme.”; “I choose to use Cantonese or English to post messages, and mainly use Cantonese as the language when making posts.” This boundary work manifested not only in linguistic practices but also in content selection and adherence to interaction norms, resonating with He et al.’s (2009) findings on language as a critical variable in social integration and Kwok-Bun and Peverelli’s (2013) observations on structural discrimination mechanisms in Hong Kong.

Bridging Roles emerged as a positive facilitator of interactions. Some long-term Mainland immigrants in Hong Kong assumed the role of cultural bridges on the platform, promoting intergroup understanding by translating cultural meanings and moderating conflictual discourse. These bridgers typically exhibited traits associated with the “Cross-Perspective Thinking” category, as one interviewee articulated: “Actually, I’m not someone who particularly likes to post things online. For example, if I see a post where there is a war of words between Mainland people and Hong Kong people in the comments, I might take a look. Whichever side, whether it’s the Mainland people or the Hong Kong people, makes sense, I will give them a thumbs-up. If the Mainland people make sense, I’ll give them a thumbs-up, and if the Hong Kong people make sense, I’ll do the same for them. That is, I stand by the truth, not by my kin. I think this is very important. People should have a dialectical way of thinking and not let their stance determine their way of thinking.” This phenomenon closely aligns with Putnam’s (2000) concept of “bridging social capital” in social capital theory.

Interaction Evolution Trajectories exhibited dynamic and nonlinear characteristics. Intergroup relations evolved with deepening interactions, transitioning from initial stereotypes and defensive interactions to more complex mutual understanding or, conversely, reinforced divisions. Interview data suggested that positive cross-group interactions often fostered bicultural identity development, while negative experiences could reinforce separation or marginalization strategies. Interviewees noted shifts in the platform’s discursive climate: “A few years ago, when I browsed LIHKG, I felt that there were more negative news about the Chinese Mainland. But now, I think there are more and more positive news about it.” This finding extends Allport et al.’s (1954) contact hypothesis, emphasizing that online contact similarly influences intergroup relations, though its impact is moderated by platform characteristics and interaction quality.

Digital Platform Characteristics profoundly affected interaction patterns. LIHKG’s anonymity mechanisms, upvote/downvote system, and forum segmentation created a unique interaction environment, directly linked to the categories of “Digital Anonymity Effects” and “Platform Structural Bias.” Interviewees observed: “Then on the LIHKG platform, if you act as a keyboard warrior, that is, once you connect the network cable and then when you unplug it, no one can see you. Then you will surely say whatever you want. I think this is quite obvious in Hong Kong.” Liang and Lee’s (2021) research highlights LIHKG’s user-voting-driven anonymity and “thread popularity” system. This study further found out that these mechanisms both facilitated authentic emotional expression and potentially amplified polarized discourse. The upvote/downvote system created a visible social endorsement mechanism, influencing content visibility and posting strategies. These findings suggest that platform design is not merely a neutral communication tool but an active structural force shaping interaction patterns and identity expression.

## 7 Research conclusion and contributions

### 7.1 Summary of research findings

By employing a grounded theory approach, this study systematically analyzed the identity negotiation processes of Mainland Chinese immigrants on the LIHKG platform. Drawing on the data analysis and theoretical constructs presented in Chapter 5, the study distilled four core findings that illuminate the central role of place in digital identity construction.

The Multifaceted Strategic Nature of Identity Negotiation emerged as the primary finding. Mainland Chinese immigrants exhibited a diverse array of identity strategies, ranging from assimilation, confrontation, and negotiation to avoidance approaches, reflecting the initiative and flexibility inherent in identity construction across multiple place contexts. Open and axial coding in Chapter 5 identified several categories—such as “Identity Duality Awareness,” “Identity Position Fluidity,” and “Identity Performance Strategies”—that directly supported this finding. Significantly, these strategies were consistently organized around place-based categories, with immigrants strategically emphasizing or de-emphasizing their connections to Mainland China as place of origin versus Hong Kong as place of residence depending on contextual demands. Different from to the linear adaptation models emphasized in traditional migration studies, this study revealed the highly strategic and context-dependent nature of identity negotiation in digital environments where virtual place-making practices intersect with physical place-based identities. These strategies were shaped by individual factors, platform structures, and sociopolitical contexts, resonating with Ellis and Bhatia's (2018) view that immigrant identities are the dynamic outcome of ongoing negotiations between individuals and their social environments. Notably, the study demonstrated that immigrants flexibly adjusted their identity expression strategies based on specific topical domains and interaction partners, aligning with Liang and Lee's (2021) observations on topic differentiation within the LIHKG platform as a place-specific digital environment.

The Central Role of the Emotional Dimension in Identity Negotiation constituted the second key finding. Categories such as “Internalization and Resistance to Exclusion,” “Emotional Defense Mechanisms,” and “Self-constrained Emotional Expression” in Chapter 5 directly supported this insight. Emotions emerged as fundamentally place-based, with participants expressing distinct emotional responses to different spatial contexts—nostalgia for Mainland places of origin, anxiety about Hong Kong as place of destination, and complex feelings about LIHKG as digital place of belonging. Emotions served not only as a channel for expressing adaptation challenges but also as a strategic tool for forging social connections and group identification across place boundaries. Through analysis of posts and interviews, the study found that shifts in emotional expression patterns often foreshadowed identity transformations particularly as immigrants developed new place-based attachments, while the formation of emotional communities provided critical adaptation support for immigrants. This finding challenges the traditional oversight of emotional factors in migration studies, highlighting emotions as a core mediating mechanism in cognition, social interaction, and identity construction within complex place configurations. It extends Mo et al.'s (2006) perspective on the emotional challenges faced by Chinese immigrants in Hong Kong by demonstrating how these challenges are fundamentally organized around contested place-based belongings.

Topic-Specific and Context-Dependent Interactions represented the third core finding, directly tied to categories such as “Cross-boundary Professional Interaction,” “Avoidance of Political Discourse,” and “Platform Space Exclusion.” These interaction patterns revealed how place operates as an organizing principle for social engagement, with different topical domains creating distinct place-based boundary dynamics. Interactions between Mainland Chinese immigrants and local residents exhibited pronounced topic differentiation, with distinct boundary negotiation practices across thematic domains. Political topics heightened place-based exclusions (Mainland versus Hong Kong), while professional and everyday life topics created spaces for place-transcendent collaboration. Group boundaries were reinforced in politically sensitive topics, whereas interactions in everyday life and professional spheres were more inclusive and collaborative. These interactions not only reflected existing social divisions but also created potential spaces for cross-group understanding through the strategic deployment of place-neutral interaction strategies. Ong and Lin's (2017) research on Hong Kong-Mainland “othering” highlighted the unique context of this phenomenon, while this study further elucidated its differential manifestations of othering across topical domains as these relate to different place-based identity categories, aligning with Leung et al.'s (2022) exploration of LIHKG’s diverse topical spaces.

The Profound Shaping Role of Digital Platform Characteristics in identity negotiation formed the fourth key finding, directly linked to categories such as “Digital Anonymity Effect” and “Platform Structural Bias.” LIHKG emerged as a distinctive digital place with its own spatial logic, community norms, and place-making practices that both reflected and reshaped offline Hong Kong place-based identities. LIHKG’s anonymity, evaluation mechanisms, and community norms provided a unique space for identity experimentation, unavailable in offline interactions, while also potentially amplifying certain group stereotypes rooted in place-based categories. Erni and Zhang (2022) described LIHKG as a “wild” haven for political expression, and this study further revealed how these platform characteristics influenced immigrants’ identity expression and interaction strategies within this specific digital place context. The findings underscored that platform design is not a neutral carrier for identity expression but an active structural force shaping the identity negotiation process through place-specific digital affordances. Data indicated that anonymity facilitated authentic emotional expression but weakened social normative constraints, contributing to the emergence of extreme discourse often organized around exclusionary place-based categories. This aligns with Matamoros-Fernández and Farkas's (2021) research on online hate speech.

### 7.2 Theoretical contributions

This study extends existing theoretical frameworks in three key dimensions while foregrounding the central analytical importance of place:

The Place-Centered Digital Expansion of Immigrant Identity Theory represents the primary theoretical contribution. By conceptualizing identity negotiation as fundamentally organized around multiple intersecting place dimensions—physical Hong Kong, digital LIHKG space, and imagined cultural territories—this study transcends both the geographical constraints of traditional immigrant identity theories and the placeless assumptions of much digital media research. The findings demonstrate that place operates not merely as context but as constitutive element in identity construction, with immigrants strategically deploying place-based categories and affiliations as identity resources.

Traditional Western migration theories, particularly those developed in North American and European contexts, often assume universal patterns of cultural adaptation based on binary assimilation-separation frameworks and linear temporal progressions (Portes and Rumbaut, 2014). These universalist assumptions include: (1) the presumption that identity adaptation follows predictable stages regardless of specific cultural contexts and place-based configurations; (2) the emphasis on eventual convergence toward host society norms as the optimal outcome without attention to how place-based distinctions may persistently organize social life; and (3) the treatment of digital spaces as merely supplementary to offline integration processes rather than as distinct identity construction arenas with their own place-based logics.

Our findings directly challenge these assumptions by demonstrating that identity negotiation in the Hong Kong-Mainland context exhibits fundamentally different dynamics due to the unique “One Country, Two Systems” framework, shared cultural heritage combined with political differentiation, and the central role of digital platforms in identity experimentation all of which create distinctive place-based identity possibilities. The four negotiation patterns identified (integrative, confrontational, collaborative, avoidance) do not follow linear progression but represent strategic repertoires deployed contextually across different place contexts, contradicting stage-based adaptation models. Furthermore, rather than convergence toward a single host identity, participants demonstrated sustained hybrid positioning that leverages multiple identity resources simultaneously organized around complex place-based affiliations.

The Integration of Emotional Sociology with Migration Studies constitutes the second theoretical innovation with particular attention to the place-based dimensions of emotional experience. This study systematically introduces the perspective of emotional sociology into migration adaptation research, highlighting the central role of emotions as a resource for identity construction across multiple place contexts. By developing an “Emotion-right Negotiation” analytical framework, the study clarifies the dialectical relationship between emotional expression, emotion management, and identity formation as these unfold within specific place-based configurations, addressing a theoretical gap in the emotional dimension of migration studies. The research reveals how emotions are fundamentally place-bound, with distinct emotional repertoires associated with different spatial contexts and place-based identity positions. This integration resonates with Svašek's (2010) research on the emotional experiences of migrants, offering a more subtle theoretical lens for understanding the emotional basis of migrant experiences within complex place systems. Categories such as “Emotional Defense Mechanisms,” “Conflict Avoidance Techniques,” and “Rationalized Coping Strategies” form the foundation of this theoretical framework while demonstrating consistent place-based organization.

Localized Place-Based Theory-Building in the Hong Kong-Mainland Context represents the third significant contribution. The study demonstrates how the unique spatial configuration of Hong Kong-Mainland relations—characterized by political autonomy within national unity, cultural similarity with systemic difference, and geographical proximity with social distance—creates distinctive place-based identity dynamics that cannot be captured by universalist migration theories. The “One Country, Two Systems” framework creates what we term “nested place identities” where immigrants must navigate belonging to multiple, partially overlapping political and cultural territories simultaneously.

Grounded in Hong Kong’s unique socio-political context, the study constructs a localized theoretical model of immigrant identity negotiation, challenging the universalist assumptions of Western migration theories described above. By illuminating the distinctive complexity of identity under the “One Country, Two Systems” framework as a unique place-based political arrangement, the study enriches the cultural diversity of migration research and provides new insights into identity politics in non-Western contexts where place boundaries may not align with national boundaries. This contribution responds to Ngo and Li's (2016) call for research on immigrant cultural identity and adaptation in the Hong Kong context, advancing a more contextually grounded theoretical perspective that accounts for specific historical, political, and cultural configurations and their distinctive place-based dynamics rather than assuming universal applicability of Western-derived models.

### 7.3 Practical implications

#### 7.3.1 Place-sensitive policy development and implementation

Study findings highlight the critical importance of developing place-sensitive approaches to digital inclusion and immigrant integration. Policies must account for the multiple place dimensions that shape immigrant experiences—physical location within Hong Kong’s specific political and cultural context, digital platform spaces with their own place-based norms, and the complex place-based identity negotiations these create.

#### 7.3.2 Study limitations and sampling considerations

Several limitations should be acknowledged in interpreting these findings. First, our sampling strategy focusing on high-engagement posts (300 + likes/dislikes, 20 + replies) may overrepresent controversial or conflict-heavy discussions compared to typical LIHKG discourse about Mainland Chinese immigrants. This selection bias toward polarized content could amplify certain identity negotiation strategies while potentially underrepresenting more moderate or everyday identity expressions. However, this approach was methodologically justified as high-engagement posts provide richer material for analyzing substantive identity negotiations and capture moments where identity boundaries become most visible and contested particularly around place-based categories.

Second, our interview sample, while diverse in regional and occupational backgrounds, represents individuals with sustained LIHKG engagement and may not capture perspectives of immigrants who avoid online political spaces entirely. Future research should examine identity negotiation among immigrants with different digital media usage patterns and varying levels of platform engagement across different place contexts.

Third, the temporal scope of our data collection (2018–2025) encompasses significant political changes in Hong Kong, including the 2019 protests and subsequent legislative developments. These macro-level changes undoubtedly influenced platform discourse patterns and individual identity strategies, suggesting that our findings are historically situated rather than universally applicable across different political periods and place-based configurations.

The Development of Digital Inclusion Policies represents the primary practical implication requiring careful attention to place-based dimensions. The study underscores digital inclusion as a critical dimension of social integration, recommending that policymakers incorporate digital inclusivity into immigrant integration frameworks that account for the multiple place contexts immigrants navigate. Specific measures include: (1) establishing digital literacy training programs for immigrants to enhance their effective use of digital platforms for social interaction across different place-based community contexts; (2) developing intercultural digital dialogue platforms to create safe communication spaces for diverse groups that can bridge place-based divisions; and (3) launching online cultural exchange initiatives to foster mutual understanding among groups through place-transcendent shared activities. These recommendations align with Leurs's (2020) findings on the role of digital platforms in supporting marginalized immigrants’ adaptation to the information society, while being empirically grounded in the categories of “Construction of Intercultural Dialogue” and “Strategies for Linguistic Capital Accumulation” identified in this study as place-sensitive practices.

Platform Governance Practices constitute the second key implication domain requiring recognition of platforms as distinctive place-based environments. Drawing on findings related to “Platform Structural Bias” and “Digital Anonymity Effect,” the study proposes platform design and governance strategies to promote healthy cross-group interactions that can transcend exclusionary place-based boundaries. These include: (1) optimizing content recommendation algorithms to mitigate information cocoons and polarization particularly around place-based identity categories; (2) developing incentive mechanisms for positive interactions to encourage constructive dialogue across place-based differences; and (3) establishing effective systems for identifying and intervening in hate speech while safeguarding freedom of expression with particular attention to place-based hate speech patterns. These suggestions address concerns raised by Matamoros-Fernández and Farkas (2021) regarding the impact of online hate speech on social cohesion and resonate with Leung et al.'s (2022) research on the influence of platform voting mechanisms on user interactions within place-specific digital environments.

Enhancement of Immigrant Support Services represents the third practical domain, directly informed by observations related to “Tendency Toward Homogeneous Interaction” and “Strategies for Linguistic Capital Accumulation.” Services must be designed to address the complex place-based challenges immigrants face as they navigate between different spatial contexts and place-based identity categories. The study recommends developing targeted digital literacy training and online social support networks to assist new immigrants in leveraging digital platforms for cultural adaptation across multiple place dimensions. Specific proposals include: (1) organizing online peer support groups to facilitate experience sharing that can help immigrants navigate place-based transitions; (2) providing guidance on platform-specific cultural norms as these vary across different digital places; and (3) creating digital immigrant resource platforms to integrate information and services organized around both Hong Kong place-specific needs and broader immigrant experiences. These measures align with Shao's (2009) suggestions, based on the “uses and gratifications” theory, to enhance immigrants’ emotional expression capacities.

Public Discourse Construction is the fourth significant implication requiring attention to how place-based categories organize public conversation. Based on the analysis of “Identity Performance Strategies” and “Dilemma of the Identity Marginalization,” the study advocates for fostering a more inclusive public discourse environment to reduce stereotypes about immigrant groups and promote multicultural understanding through place-transcendent narratives. Recommendations include: (1) diversifying media reports of Mainland immigrants’ contributions and experiences that move beyond exclusionary place-based framings; (2) promoting intercultural dialogue initiatives to create shared narratives that can bridge place-based divisions; and (3) developing community engagement activities rooted in common interests that transcend place-based boundaries. These suggestions resonate with Van Eldik et al.'s (2019) research on “mediated urban belonging,” emphasizing the reinforcement of offline identity negotiation and group interaction experiences through online content.

The Hong Kong-Mainland migration context offers crucial insights for understanding how place operates as both constraint and resource in contemporary identity construction processes. As global migration increasingly involves movement between places with complex political relationships and as digital technologies create new forms of virtual place-making, the theoretical and practical insights from this study become relevant far beyond the specific Hong Kong context. Future research should continue exploring how place-based identity dynamics unfold across different migration contexts and digital platforms, contributing to more nuanced understandings of identity, belonging, and integration in our increasingly complex spatial world.

Through continued exploration of these research directions, future studies will further deepen the understanding of immigrant identity construction and cultural adaptation in the digital era, providing a more robust theoretical and practical foundation for promoting social integration across multiple place dimensions. In particular, the findings from the Hong Kong-Mainland context within global migration research not only enrich the cultural diversity of migration studies but also offer new perspectives for understanding the increasingly complex identity politics in the context of globalization where place-based identities remain crucial organizing principles for social life.

## Data Availability

The original contributions presented in the study are included in the article/supplementary material, further inquiries can be directed to the corresponding author.

## References

[ref1] Albrecht M. R. Blasco J. Jensen R. B. Mareková L. (2021) Collective information security in large-scale urban protests: The case of Hong Kong. 30th USENIX security symposium (USENIX security 21) (pp. 3363–3380)

[ref2] Allport G. W. Clark K. Pettigrew T. (1954). The nature of prejudice. Reading,Massachusetts,USA: Addison-Wesley.

[ref3] Appadurai A. (1996). Modernity at large: Cultural dimensions of globalization. Minneapolis,USA: University of Minnesota Press.

[ref4] Apple Daily (2019) 55% respondents: LIHKG the most critical, Apple Daily A. Hong Kong: Next Digital Limited.

[ref5] Au H. M. Chan C. C. Chan L. I. Li K. J. Ng Y. H. R. (2021). Gratification of social media during social movement: A case study of LIHKG usage by" read-only" users during the 2019 anti-extradition bill movement in Hong Kong. Hong Kong: The University of Hong Kong.

[ref6] Berry J. W. (2005). Acculturation: living successfully in two cultures. Int. J. Intercult. Relat. 29, 697–712. doi: 10.1016/j.ijintrel.2005.07.013

[ref7] Bhabha H. K. (1994). The location of culture. Abingdon, Oxfordshire, UK: Routledge.

[ref8] Bhatia A. (2016). “Occupy central” and the rise of discursive illusions: a discourse analytical study. Text Talk 36, 661–682. doi: 10.1515/text-2016-0029

[ref9] Bhatia S. Ram A. (2009). Theorizing identity in transnational and diaspora cultures: a critical approach to acculturation. Int. J. Intercult. Relat. 33, 140–149. doi: 10.1016/j.ijintrel.2008.12.009

[ref10] Boyd D. (2014). It's complicated: The social lives of networked teens. New Haven, Connecticut, USA: Yale University Press.

[ref11] Brinkerhoff J. M. (2009). Digital diasporas: Identity and transnational engagement. Cambridge, UK: Cambridge University Press.

[ref12] Bucholtz M. Hall K. (2005). Identity and interaction: a sociocultural linguistic approach. Discourse Stud. 7, 585–614. doi: 10.1177/1461445605054407

[ref13] Cao B. Chen Z. Huang Y. Lo W. H. (2014). Conflict between mainland Chinese and Hong Kongers: a social identity perspective in explaining the hostile media phenomenon and the third-person effect. J. Appl. Journal. Media Stud. 3, 225–240. doi: 10.1386/ajms.3.2.225_1

[ref14] Census and Statistics Department (2025) Table 110–01003: Population growth by component. Government of the Hong Kong Special Administrative Region.

[ref15] Chan M. (2015). Psychological antecedents and motivational models of collective action: examining the role of perceived effectiveness in political protest participation. Soc. Mov. Stud. 15, 305–321. doi: 10.1080/14742837.2015.1096192

[ref17] Charmaz K. (2014). Constructing grounded theory. 2nd Edn. London, UK: Sage Publications.

[ref18] Ching S. Hsiao C. Wan S. K. (2012). Impact of CEPA on the labor market of Hong Kong. China Econ. Rev. 23, 975–981. doi: 10.1016/j.chieco.2012.04.017

[ref19] Chiu S. W. K. Choi S. Y. P. Ting K. F. (2005). Getting ahead in the capitalist paradise: migration from China and socioeconomic attainment in colonial Hong Kong 1. Int. Migr. Rev. 39, 203–227. doi: 10.1111/j.1747-7379.2005.tb00260.x

[ref20] Chou K. L. (2013). Familial effect on child poverty in Hong Kong immigrant families. Soc. Indic. Res. 113, 183–195. doi: 10.1007/s11205-012-0088-7

[ref21] Creswell J. W. Plano Clark V. L. (2017). Designing and conducting mixed methods research. 3rd Edn. London, UK: Sage Publications.

[ref22] Diminescu D. (2008). The connected migrant: an epistemological manifesto. Soc. Sci. Inf. 47, 565–579. doi: 10.1177/0539018408096447

[ref23] Downes J. F. (2017). “Mainland Chinese immigration in Hong Kong: Analysing anti-immigrant sentiment” in Citizenship, identity and social movements in the new Hong Kong. Eds. L. Wai-man, and C. Luke (Abingdon, Oxfordshire, UK: Routledge), 51–71.

[ref24] Ellis B. D. Bhatia S. (2018). Cultural psychology for a new era of citizenship politics. Cult. Psychol. 25, 220–240. doi: 10.1177/1354067x18808760

[ref25] Erni J. N. Zhang Y. (2022). Wild hopes: sourcing the political vocabulary of digital citizenship from the LIHKG forum. Int. Commun. Gaz. 84, 349–375. doi: 10.1177/17480485221094123

[ref26] Goffman E. (1963). Stigma: Notes on the management of spoiled identity. Hoboken, New Jersey, USA: Prentice-Hall.

[ref27] Gross J. J. (2015). Emotion regulation: current status and future prospects. Psychol. Inq. 26, 1–26. doi: 10.1080/1047840X.2014.940781

[ref28] He X. Lou W. Zhao H. (2009). Optimistic orientation, social service utilization and social integration: an exploratory study of new immigrants in Hong Kong. Northwest Populat. 30, 23–26.

[ref29] Hermans H. J. M. Dimaggio G. (2007). Self, identity, and globalization in times of uncertainty: a dialogical analysis. Rev. Gen. Psychol. 11, 31–61. doi: 10.1037/1089-2680.11.1.31

[ref30] Hong Kong Census and Statistics Department. (2021-2024). Available online at: https://www.censtatd.gov.hk/tc/ (Accessed March 23, 2025).

[ref31] Immigration Department (2024) Available online at: https://www.immd.gov.hk/hkt (Accessed March 21, 2025).

[ref32] Ju R. Hamilton L. McLarnon M. (2021). The medium is the message: WeChat, YouTube, and Facebook usage and acculturation outcomes. Int. J. Commun. 15:23.

[ref33] King R. Wood N. (2010). Media and migration. London, UK: Taylor and Francis.

[ref34] Komito L. (2011). Social media and migration: virtual community 2.0. J. Am. Soc. Inf. Sci. Technol. 62, 1075–1086. doi: 10.1002/asi.21517

[ref35] Kozinets R. V. (2020). Netnography: The essential guide to qualitative social media research. 3rd Edn. London, UK: Sage Publications.

[ref36] Kwok-Bun C. Peverelli P. J. (2013). “Hybridity and its discontents: strategies of adaptation of Hong Kong’s professional immigrants from mainland China” in Routledge eBooks, ed. Kwok-bun C. London, UK: Routledge. 40–74.

[ref37] Lam J. T. M. (2015). Political decay in Hong Kong after the occupy central movement. Asian Aff. Am. Rev. 42, 99–121. doi: 10.1080/00927678.2015.1035143

[ref38] Law K. Y. Lee K. M. (2006). Citizenship, economy and social exclusion of mainland Chinese immigrants in Hong Kong. J. Contemp. Asia 36, 217–242. doi: 10.1080/00472330680000131

[ref39] Lecheler S. Matthes J. Boomgaarden H. (2019). Setting the agenda for research on media and migration: state-of-the-art and directions for future research. Mass Commun. Soc. 22, 691–707. doi: 10.1080/15205436.2019.1688059

[ref40] Lee F. L. F. Cheng E. W. Liang H. Tang G. K. Y. Yuen S. (2021). Dynamics of tactical radicalisation and public receptiveness in Hong Kong’s anti-extradition bill movement. J. Contemp. Asia 52, 429–451. doi: 10.1080/00472336.2021.1910330

[ref41] Lee S. Y. Ng I. F. S. Chou K. L. (2015). Exclusionary attitudes toward the allocation of welfare benefits to Chinese immigrants in Hong Kong. Asian Pac. Migr. J. 25, 41–61. doi: 10.1177/0117196815619805

[ref42] Lee L. H. Tsui W. K. (2020). Deep learning on social media discussion. Available online at: https://lhlee99.github.io/FYP-webpage/documents/ProjectPlan.pdf (Accessed February 02, 2025).

[ref43] Leudar I. Marsland V. Nekvapil J. (2004). On membership categorization: ‘us’,‘them’and ‘doing violence’in political discourse. Discourse Soc. 15, 243–266. doi: 10.1177/0957926504041019

[ref44] Leung B. Hsiao Y. Garimella K. (2022). Decentralized yet unifying: digital media and solidarity in Hong Kong's anti-extradition movement. J. Quant. Descript. 2:17. doi: 10.51685/jqd.2022.017

[ref45] Leurs K. (2020). “Migration infrastructures” in The SAGE handbook of media and migration. Eds. K. Smets, K. Leurs, M. Georgiou, and S. Witteborn. (London, UK: Sage Publications), 91–102.

[ref46] Leurs K. Prabhakar M. (2018). Doing digital migration studies: Methodological considerations for an emerging research focus. Cham, Switzerland: Qualitative Research in European Migration Studies, 247–266.

[ref47] Liang H. Lee F. L. F. (2021). Thread popularity inequality as an indicator of organization through communication in a networked movement: an analysis of the LIHKG forum. Chinese J. Communic. 15, 332–354. doi: 10.1080/17544750.2021.1922475

[ref48] Lim S. S. Pham B. (2016). ‘If you are a foreigner in a foreign country, you stick together’: technologically mediated communication and acculturation of migrant students. New Media Soc. 18, 2171–2188. doi: 10.1177/1461444816655612

[ref49] Ma A. Holford J. (2023). Mainland Chinese students in Hong Kong: coping with the socio-political challenges of 2017 to 2020. J. Stud. Int. Educ. 28, 588–605. doi: 10.1177/10283153231187142

[ref50] Matamoros-Fernández A. Farkas J. (2021). Racism, hate speech, and social media: a systematic review and critique. Televi. New Media 22, 205–224. doi: 10.1177/1527476420982230

[ref51] Matthew C. M. The translocalism of Hong Kong popular culture: an analysis of a critical internet meme co-created across Hong Kong and China. Hong Kong Studies, (2021) Available online at: https://cup.cuhk.edu.hk/image/catalog/journal/jpreview/HKS3.1.01.pdf (Accessed February 13, 2025).

[ref52] McGregor E. Siegel M. (2013). Social media and migration research. Maastricht, Netherlands: UNU Migration Network.

[ref53] Mo P. K. H. Mak W. W. S. Kwan C. S. Y. (2006). Cultural change and Chinese immigrants' distress and help-seeking in Hong Kong. J. Ethn. Cult. Divers. Soc. Work. 15, 129–151. doi: 10.1300/j051v15n03_06

[ref54] Nedelcu M. (2012). Migrants' new transnational habitus: rethinking migration through a cosmopolitan lens in the digital age. J. Ethn. Migr. Stud. 38, 1339–1356. doi: 10.1080/1369183x.2012.698203

[ref55] Ngo H. Y. Li H. (2016). Cultural identity and adaptation of mainland Chinese immigrants in Hong Kong. Am. Behav. Sci. 60, 730–749. doi: 10.1177/0002764216632837

[ref56] Oksanen A. Hawdon J. Holkeri E. Näsi M. Räsänen P. (2014). “Exposure to online hate among young social media users” in Soul of society: A focus on the lives of Children & Youth (Bingley, UK: Emerald Group Publishing Limited), 253–273.

[ref57] Ong J. C. Lin T. Z. (2017). “Plague in the city: digital media as shaming apparatus toward mainland Chinese ‘locusts’ in Hong Kong” in Communicating the City: Meanings, practices, interactions. ed. Lang P. (New York, USA: Peter Lang), 149–164.

[ref58] Patton M. Q. (2015). Qualitative research and evaluation methods. 4th Edn. Thousand Oaks, California, USA: Sage Publications.

[ref59] Peng Y. (2016). Student migration and polymedia: mainland Chinese students’ communication media use in Hong Kong. J. Ethn. Migr. Stud. 42, 2395–2412. doi: 10.1080/1369183x.2016.1194743

[ref60] Phinney J. S. (2003). “Ethic identity and acculturation” in Acculturation: Advances in theory, measurement, and applied research (Washington, DC, USA: American Psychological Association), 63–81.

[ref61] Ponzanesi S. Leurs K. (2022). Digital migration practices and the everyday. Communic. Culture Critique 15, 103–121. doi: 10.1093/ccc/tcac016

[ref62] Portes A. Rumbaut R. G. (2014). Immigrant America: A portrait. 4th Edn. Oakland, California, USA: University of California Press.

[ref63] Putnam R. D. (2000). Bowling alone: The collapse and revival of American community. New York, NY, USA: Simon and schuster.

[ref64] Qiu Y. (2020). Chinese digital diasporic media and the shaping of identity: The case of UKzone. Lund, Sweden: Lund University.

[ref65] Shao G. (2009). Understanding the appeal of user-generated media: a uses and gratification perspective. Internet Res. 19, 7–25. doi: 10.1108/10662240910927795

[ref66] So A. Y. Ip P. L. (2019). Civic localism, anti-mainland localism, and independence: the changing pattern of identity politics in Hong Kong special administrative region. Asian Educ. Dev. Stud. 9, 255–267. doi: 10.1108/aeds-02-2018-0043

[ref67] Song Y. Wu Y. (2018). Tracking the viral spread of incivility on social networking sites: the case of cursing in online discussions of Hong Kong–mainland China conflict. Commun. Public 3, 46–61. doi: 10.1177/2057047318756408

[ref68] Stekelenburg J. Klandermans B. (2013). Fitting demand and supply: how identification brings appeals and motives together. Soc. Mov. Stud. 13, 179–203. doi: 10.1080/14742837.2013.843448

[ref69] Strauss A. Corbin J. (1998). Basics of qualitative research: Techniques and procedures for developing grounded theory. 2nd Edn. Thousand Oaks, California, USA: Sage Publications.

[ref70] Svašek M. (2010). On the move: emotions and human mobility. J. Ethn. Migr. Stud. 36, 865–880. doi: 10.1080/13691831003643322

[ref71] Ting T. Y. (2024). The pitfalls of smart urban infrastructure during a period of unrest: networked dissent against smart lampposts in Hong Kong. J. Infrastruct. Policy Dev. 8:8313. doi: 10.24294/jipd.v8i9.8313

[ref72] Tsang G. F. Y. (2020). Parochial apolitical formulation: Hong Kong internetization and the sexualizing cyberspace of the storytelling channels of the Golden forum and the LIHKG forum. Sungkyun J. East Asian Stud. 20, 61–82. doi: 10.21866/esjeas.2020.20.3.003

[ref73] Turner J. C. Tajfel H. (1986). “The social identity theory of intergroup behavior” in Psychology of intergroup relations, vol. 5. Eds. S. Worchel, and W. G. Austin. (Chicago, Illinois, USA: Nelson-Hall), 7–24.

[ref74] Van Eldik A. K. Kneer J. Lutkenhaus R. O. Jansz J. (2019). Urban influencers: an analysis of urban identity in YouTube content of local social media influencers in a super-diverse city. Front. Psychol. 10:2876. doi: 10.3389/fpsyg.2019.02876, PMID: 31920892 PMC6930894

[ref75] Veg S. (2017). The rise of “localism” and civic identity in post-handover Hong Kong: questioning the Chinese nation-state. China Q. 230, 323–347. doi: 10.1017/s0305741017000571

[ref76] Wang C. (2012). Bridging borders in the global city: negotiating sameness and difference in Hong Kong’s skilled immigrants from mainland China. J. Int. Migr. Integr. 13, 565–581. doi: 10.1007/s12134-012-0236-6

[ref77] Wong W. K. F. Chou K. L. Chow N. W. S. (2011). Correlates of quality of life in new migrants to Hong Kong from mainland China. Soc. Indic. Res. 107, 373–391. doi: 10.1007/s11205-011-9853-2, PMID: 22611300 PMC3342488

[ref78] Wong K. T. W. Zheng V. Wan P. S. (2016). The impact of cross-border integration with mainland China on Hong Kong's local politics: the individual visit scheme as a double-edged sword for political trust in Hong Kong. China Q. 228, 1081–1104. doi: 10.1017/s0305741016001478

[ref79] Yang D. M. H. (2016). Book review: identity, hybridity, and cultural home: Chinese migrants and diaspora in multicultural societies. Int. Migr. Rev. 50, e56–e57. doi: 10.1111/imre.12275

[ref80] Yang C. Li S.-m. (2013). Transformation of cross-boundary governance in the greater Pearl River Delta, China: contested geopolitics and emerging conflicts. Habitat Int. 40, 25–34. doi: 10.1016/j.habitatint.2013.02.001, PMID: 32287700 PMC7115696

[ref81] Yau A. Marder B. O’Donohoe S. (2019). The role of social media in negotiating identity during the process of acculturation. Inf. Technol. People 33, 554–575. doi: 10.1108/itp-09-2017-0305

[ref82] Yeo R. (2019). ExplainerHong Kong protests: How the city’s reddit-like forum LIHKG has become the leading platform for organising demonstrations. South China Morning Post. Available online at: https://www.scmp.com/news/hong-kong/society/article/3021224/hong-kong-protests-how-citys-reddit-forum-lihkg-has-become (Accessed February 11, 2025).

[ref83] Yu X. Stewart S. M. Liu I. K. F. Lam T. H. (2013). Resilience and depressive symptoms in mainland Chinese immigrants to Hong Kong. Soc. Psychiatry Psychiatr. Epidemiol. 49, 241–249. doi: 10.1007/s00127-013-0733-8, PMID: 23818045

[ref84] Yu C. Tay D. Jin Y. Yuan X. (2023). Speech acts and the communicative functions of emojis in LIHKG online discussion forum amid COVID-19. Front. Psychol. 14:1207302. doi: 10.3389/fpsyg.2023.1207302, PMID: 37496797 PMC10366367

[ref85] Yuen S. Chung S. (2018). Explaining localism in post-handover Hong Kong: an eventful approach. China Perspect. 2018, 19–29. doi: 10.4000/chinaperspectives.8044

[ref86] Zhang Z. Wu X. (2011). Social change, cohort quality and economic adaptation of Chinese immigrants in Hong Kong, 1991–2006. Asian Pac. Migr. J. 20, 1–29. doi: 10.1177/011719681102000101

[ref87] Zhu Y. (2015). Brokering identity and learning citizenship: immigration settlement organizations and new Chinese immigrants in Canada. J. Soc. Sci. Educ. 14, 9–19. doi: 10.2390/jsse-v14-i3-1403

[ref88] Zhu A. Y. F. Chou K. L. (2021). Collective action in the anti-extradition Law amendment bill movement in Hong Kong: two integrative group identification models. Anal. Soc. Issues Public Policy 21, 1033–1053. doi: 10.1111/asap.12268

[ref89] Zimmer M. (2018). Addressing conceptual gaps in big data research ethics: an application of contextual integrity. Soc. Media Soc. 4, 1–11. doi: 10.1177/2056305118768300

